# Assessing the decision quality of artificial intelligence and oncologists of different experience in different regions in breast cancer treatment

**DOI:** 10.3389/fonc.2023.1152013

**Published:** 2023-06-09

**Authors:** Chunguang Han, Yubo Pan, Chang Liu, Xiaowei Yang, Jianbin Li, Kun Wang, Zhengkui Sun, Hui Liu, Gongsheng Jin, Fang Fang, Xiaofeng Pan, Tong Tang, Xiao Chen, Shiyong Pang, Li Ma, Xiaodong Wang, Yun Ren, Mengyou Liu, Feng Liu, Mengxue Jiang, Jiqi Zhao, Chenyang Lu, Zhengdong Lu, Dongjing Gao, Zefei Jiang, Jing Pei

**Affiliations:** ^1^ Department of Pediatric Surgery, The First Affiliated Hospital of Anhui Medical University, Hefei, China; ^2^ Department of General Surgery, The First Affiliated Hospital of Anhui Medical University, Hefei, China; ^3^ Department of Breast Surgery, The First Affiliated Hospital of Anhui Medical University, Hefei, China; ^4^ Department of Breast Cancer, Fifth Medical Center, Chinese People’s Liberation Army General Hospital, Beijing, China; ^5^ Department of Breast Cancer, Cancer Center, Guangdong Provincial People's Hospital and Guangdong Academy of Medical Sciences, Guangzhou, China; ^6^ Department of Breast Oncology Surgery, Jiangxi Cancer Hospital (The Second People's Hospital of Jiangxi Province), Nanchang, China; ^7^ Department of Breast Surgery, Henan Provincial People's Hospital, Zhengzhou, China; ^8^ Department of Oncological Surgery, the First Affiliated Hospital of Bengbu Medical College, Bengbu, China; ^9^ Department of Thyroid and Breast surgery, the First Affiliated Hospital of Wannan Medical College (Yijishan Hospital), Wuhhu, China; ^10^ Department of General Surgury, The Second Affiliated Hospital of Anhui Medical University, Hefei, China; ^11^ Department of General Surgery, Lu'an People's Hospital of Anhui Province (Lu'an Hospital of Anhui Medical University), Lu'an, China; ^12^ Department of Thyroid and Breast Surgery, Anqing Municipal Hospital (Anqing Hospital Affiliated to Anhui Medical University), Anqing, China; ^13^ Department of Thyroid and Breast Surgery, The people's hospital of Bozhou (Bozhou Hospital Affiliated to Anhui Medical University), Bozhou, China; ^14^ Department of Thyroid and Breast surgery, Department of Oncological Surgery, Taihe county people's hospital (The Taihe hospital of Wannan Medical College), Fuyang, China; ^15^ Department of Thyroid and Breast surgery, Lixin County People's Hospital, Bozhou, China; ^16^ Department of Breast Surgery, Fuyang Cancer Hospital, Fuyang, China; ^17^ Department of Breast Surgery, Department of General Surgery, The First Affiliated Hospital of Anhui Medical University, Hefei, China

**Keywords:** clinical decision support system, CSCO AI, breast cancer, artificial intelligence, decision-making quality

## Abstract

**Background:**

AI-based clinical decision support system (CDSS) has important prospects in overcoming the current informational challenges that cancer diseases faced, promoting the homogeneous development of standardized treatment among different geographical regions, and reforming the medical model. However, there are still a lack of relevant indicators to comprehensively assess its decision-making quality and clinical impact, which greatly limits the development of its clinical research and clinical application. This study aims to develop and application an assessment system that can comprehensively assess the decision-making quality and clinical impacts of physicians and CDSS.

**Methods:**

Enrolled adjuvant treatment decision stage early breast cancer cases were randomly assigned to different decision-making physician panels (each panel consisted of three different seniority physicians in different grades hospitals), each physician made an independent “Initial Decision” and then reviewed the CDSS report online and made a “Final Decision”. In addition, the CDSS and guideline expert groups independently review all cases and generate “CDSS Recommendations” and “Guideline Recommendations” respectively. Based on the design framework, a multi-level multi-indicator system including “Decision Concordance”, “Calibrated Concordance”, “ Decision Concordance with High-level Physician”, “Consensus Rate”, “Decision Stability”, “Guideline Conformity”, and “Calibrated Conformity” were constructed.

**Results:**

531 cases containing 2124 decision points were enrolled; 27 different seniority physicians from 10 different grades hospitals have generated 6372 decision opinions before and after referring to the “CDSS Recommendations” report respectively. Overall, the calibrated decision concordance was significantly higher for CDSS and provincial-senior physicians (80.9%) than other physicians. At the same time, CDSS has a higher “ decision concordance with high-level physician” (76.3%-91.5%) than all physicians. The CDSS had significantly higher guideline conformity than all decision-making physicians and less internal variation, with an overall guideline conformity variance of 17.5% (97.5% vs. 80.0%), a standard deviation variance of 6.6% (1.3% vs. 7.9%), and a mean difference variance of 7.8% (1.5% vs. 9.3%). In addition, provincial-middle seniority physicians had the highest decision stability (54.5%). The overall consensus rate among physicians was 64.2%.

**Conclusions:**

There are significant internal variation in the standardization treatment level of different seniority physicians in different geographical regions in the adjuvant treatment of early breast cancer. CDSS has a higher standardization treatment level than all physicians and has the potential to provide immediate decision support to physicians and have a positive impact on standardizing physicians’ treatment behaviors.

## 1 Background

According to the latest global cancer burden data released by the International Agency for Research on Cancer (IARC) in 2020, cancer is still the major disease burden in the world, in which the incidence of breast cancer has surpassed lung cancer to become the first female malignant tumor (1). Oncology therapy is facing enormous challenges globally, especially in vast countries such as China, USA, and India, which are under pressure not only in terms of the patient population, but also in terms of balancing the variation of standardized oncology treatment across different geographical regions. In addition, considering the practical dilemmas such as heavy workload of clinicians, rapidly updated clinical guidelines and huge amount of literature that is difficult to grasp in a timely manner, the standardized treatment of oncology is facing a huge informational challenge (2).

Recently, artificial intelligence has been increasingly applied in medical fields such as medical imaging diagnosis (3–9), pathology diagnosis (10–14) and supporting treatment decision-making (15, 16) and has shown great potential for clinical applications. Clinical decision support systems (CDSS) for oncology, represented by Watson Oncology (WFO), are designed to overcome the informational challenges of oncology diseases and are highly expected to successfully overcome this challenge (17). CDSS can deeply analyze patients’ condition information and select the optimal treatment recommendations from the knowledge database and feedback to physicians, thus assisting them to make the most appropriate treatment programs (18, 19). A high level CDSS has great potential to overcome the current dilemmas in oncology treatment, such as standardized treatment, efficient decision-making and talent development.

CDSS require a solid clinical evidence base before they can be finally applied to the clinic, including the level of standardized treatment of CDSS and the impact on clinicians’ decision-making (19–22). However, most of the relevant clinical studies are currently limited to the stage of superficial concordance studies, in which the performance of CDSS is assessed by examining the concordance between CDSS and actual clinical treatment programs (23–37) or physician decision-making programs (27, 38); although this approach is commonly used, it lacks high-quality treatment standards and does not reflect the quality of treatment decisions (39); because the “Decision Concordance” only reflects the same situation between CDSS and physician decisions, it does not reflect whether the decisions are standardized and individualized to patients. For example, Xu F et al. (38) showed that the concordance in decision making was higher among WFOs and junior physicians than senior and middle physicians. (0.68 vs. 0.54 and 0.49, P=0.001). In addition, for the assessment of decision quality, only two studies have been reported, but they are also limited to the level of one-sided indicators such as physician empiricism (40) or simple guideline adherence (38). Xu F et al. (38) first applied “Guideline Conformity” to the study of decision quality of CDSS and physicians, but it only reflects the simple conformity between physicians’ decisions and guideline recommendations in a certain cross-section, and does not fully reflect physicians’ medical skills due to the fact that it does not take into account technical maturity. This means that a very high guideline conformity rate does not certainly indicate a high medical skill level, and usually junior physicians are more likely to adhere to guidelines at the beginning of their careers because they have not yet accumulated the same rich clinical experience as senior physicians. Thus, the current indicators, including “Decision Concordance” and “Guideline Conformity”, have obvious shortcomings in assessing the standardization treatment level of CDSS and physicians, and there is still a lack of indicator systems to comprehensively assess the standardized treatment level of physicians and CDSS, which in turn limits the in-depth research on the clinical impact of CDSS (41). In particular, it remains unknown whether CDSS-based smart medical practices can improve physicians’ standardized treatment levels and balance regional medical variations.

The aim of this study is to construct a comprehensive multi-level multi-indicator system for the assessment of the standardized treatment level of physicians and CDSS; and at the same time, to assess the current status of the standardization treatment level and internal variation in early breast cancer adjuvant treatment decision-making in different geographical regions based on this indicator system, and identified the standardized treatment level of CDSS and its clinical application potential. It also provides a methodological basis for the further construction of a sensible CDSS clinical practice model and clinical impact assessment.

## 2 Methods

### 2.1 Study design

**Figure 1:**
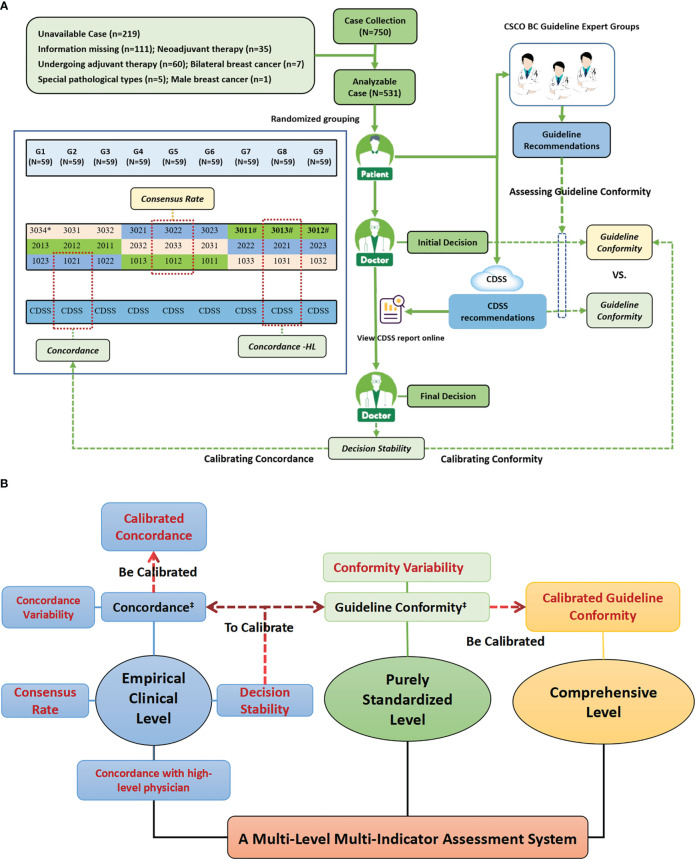
Study design profiles. **(A)** Study Flow Diagram; **(B)** The Multi-Level Multi-Indicator Assessment System Used to Comprehensively Assess the Standardization Treatment Levels of AI-based Oncology CDSS and Physicians. Figure **(A)** shows the overall workflow of the study and the generation mechanism of the primary indicators, while Figure **(B)** shows the entire multi-level, multi-indicator system that was constructed with the primary indicators as the center, which included many sub-indicators derived from the primary indicators. Figure **(A)** shows the specific composition of decision physicians and case assignment. Cases were randomly assigned to nine different case groups (G1-G9), and each case group was assigned to three different seniority physicians from different grades hospitals in the same column of the figure for decision-making, and cases were assigned to both the guideline experts group and the CDSS for independent review; for example, G1 was assigned to three physicians of 3034,2013 and 1023, as well as to the guideline experts group and the CDSS for independent review. **#** High-level physicians in this study, i.e., provincial senior physicians, included three physicians, 3011,3012 and 3013. **‡** Existing indicators reported in the current literature, while the rest indicators were constructed and applied for the first time in this study.

This is a prospective, multi-center, comparative clinical study. The design framework was illustrated in **Figure 1A**. This study protocol was approved by the ethical review committees of all hospitals involved. The study was prospective registered at Chinese Clinical Trial Registry (http://www.chictr.org.cn, ChiCTR2000039122 and ChiCTR2100047685).

### 2.2 Patient population

The cases enrolled in this study were inpatients who underwent surgical treatment and postoperative adjuvant therapy in the Department of Breast Surgery at the First Affiliated Hospital of Anhui Medical University from January to September 2020. The patients were all female, unilateral, non-specific type invasive breast cancer cases and had complete disease information without omission; others were excluded. Complete information on the condition needed to make a post-operative adjuvant treatment program is extracted from the patient’s medical record by senior researchers. The cases were randomly assigned to nine different case groups (G1-9) **(** **Figure 1A**). Informed consent was obtained from all patients in this study.

### 2.3 Composition of decision-making physicians and generation of physicians’ recommendations

We have coded different grades of hospitals, different physician seniority, and each decision-making physician independently. The numbering rules are as follows: each code consists of four digits, the first of which represents a different grades of hospital (3=provincial hospital, 2=municipal hospital, 1=county hospital), the middle two digits represent different seniority physicians (01=Senior physician, 02=middle physician, 03=junior physician), and the last digits represents the independent number within the same seniority in the same grade hospital (1-4), e.g. 3034 represents provincial hospital junior physician 4.

A total of twenty-seven physicians of different seniority (nine senior, nine middle and nine junior; 1011-3034) from ten different grade hospitals (four provincial, three municipal and three county) in Anhui province participated in the decision-making, forming a total of nine independently comparable subgroups of physicians (101-303), and each panel consisted of three physicians of the same seniority from the same level of hospital. The senior physician is the chief physician or associate chief physician with 10 years of work experience or more; the middle physician is the attending physician with 4-9 years of work experience; and the junior physician is the hospitalist with 3 years of work experience or less.

Enrolled cases were randomly assigned to three differently senior physicians from different grades of hospitals in group format (G1-9), and each physician independently reviewed all cases in the case group. For each case, the physician first makes an independent “ Initial Decision” and then reviews the CDSS report online and makes a “ Final Decision”. (**Figure 1A** **)**

In this trial, the presentation of case information, the collection of physicians’ decision-making opinions and the presentation of CDSS reports are all realized by the on-line electronic questionnaire; in particular, by setting the function of the electronic questionnaire, the physicians will directly enter the CDSS report view screen after making the “ Initial Decision “, and cannot revoke the “ Initial Decision “; any modification can only be recorded in the “ Final Decision “ collection screen. As the link shows: https://www.wjx.cn/vm/tU3PZSe.aspx.

### 2.4 CDSS and generation of AI decisions

**Figure 2:**
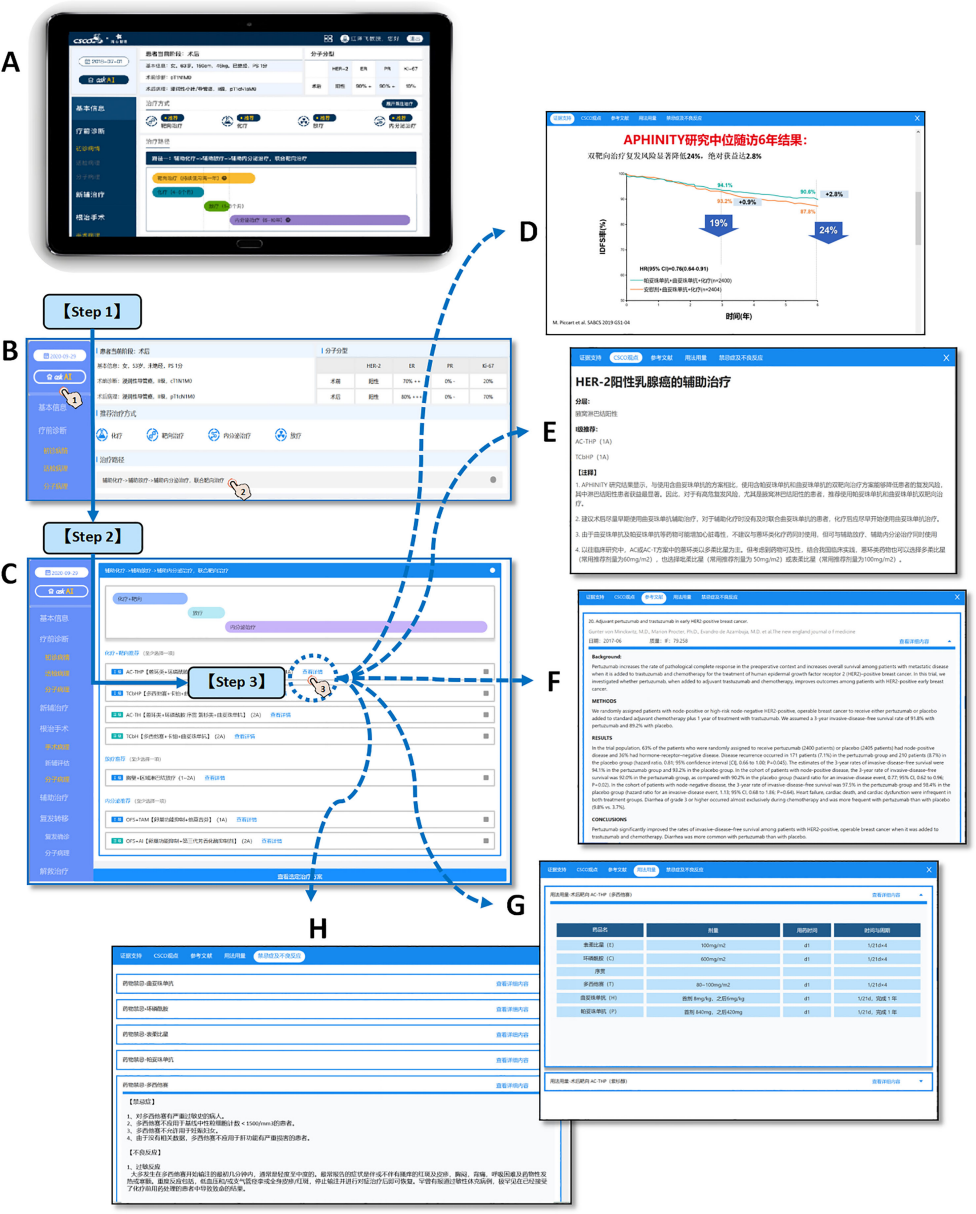
CSCO AI system structure and operation demonstration. The clinical used CSCO AI system is based on the PAD hardware system **(A)**, and the operation of making intelligent decisions for patients is as follows: [Step 1] Click “ask AI” and ask the system for treatment recommendations for the patient’s current treatment stage; CSCO AI will give the recommended treatment modality and treatment path that is appropriate for the patient’s current treatment stage **(B)**. [Step 2] Click on the treatment pathway to view the specific treatment program. For each recommended treatment modality, CSCO AI further gives specific treatment programs, including “ Level I recommendation” and “Level II recommendation”**(C)**. [Step 3] Click the “View Details” button of the specific program to further review the “Evidence Support” **(D)**, “CSCO Opinions” **(E)**, “References” **(F)**, “Dosage” **(G)** and “Contraindications and Adverse Effects” **(H)** provided by CSCO AI for the relevant recommendation; where The “Evidence Support” provides evidence-based support for the relevant programs recommended by CSCO AI; the “CSCO Opinions” provides the guideline basis for the relevant programs based on the latest CSCO BC guidelines. [Demonstration case (T571)] Patient, female, 53 years old, premenopausal; 2020-9-16 left breast lump core needle biopsy showed: invasive carcinoma, non-specific type, WHO grade 2; ER (2+,70%), PR (-), Her-2 (3+), Ki-67 (20%). 2020-9-28 left breast cancer modified radical mastectomy showed: cancer size 2·0*2·0*1·0cm; invasive carcinoma, non-specific type, WHO grade II; ER (3+,80%), PR (-), Her-2 (3+), Ki-67 (+, 70%); no clear cancer invasion of nerves and vasculature; axillary lymph nodes 1+/17. [System operation link (copy the link and open it on computer)] https://sfportal.aistarfish.com/bcresearch/show/#/csco/suggest/detail/9/BC00004370/13?phone=19912345678.

The CDSS used in this study is the Chinese Society of Clinical Oncology Artificial Intelligence System (CSCO AI, version 2021), which is an artificial intelligence-based oncology clinical decision support system developed under the platform of the Chinese Society of Clinical Oncology (CSCO) based on Chinese breast cancer data, guidelines and expert experience, and can provide treatment recommendations for physicians’ reference based on patients’ individualized conditions; it also provides treatment information such as scientific evidence, guideline basis, drug dosage, contraindications and adverse effects associated with each protocol (42). CSCO AI system structure and operation demonstration was showed in **Figure 2**.

The treatment recommendations of CSCO AI are divided into “Recommended” and “Not Recommended”, where “Recommended” is further subdivided into “Level I Recommendations” and “Level II Recommendations”. “Not Recommended” means that the patient is recommended to be exempted from the relevant treatment. “ Level I Recommendation” options are those with strong evidence and high availability, relatively stable tumor treatment value, and definite patient benefit, and are indicated in blue. “ level II Recommendation” is usually a treatment or drug that is poorly available or has a low cost-effectiveness ratio and is beyond the affordability of the population, although it is supported by high-level evidence, and is indicated in green. The specific classification criteria for the level of recommendation are shown in **Table 1**.

**Table 1:** The levels of recommendation and classification of evidence of CSCO guidelines and CSCO AI.

Part 1 Recommended Level			
Recommended Level	Classification Criteria		
**Level I Recommendation**	**Types 1A evidence and some Types 2A evidence** In general, Type 1A evidence and some Type 2A evidence (with a high level of expert consensus and good accessibility in China) are used as Level I recommendations. Specifically, level I recommendations have the following characteristics: good accessibility of universal diagnostic and treatment measures (including proof of indications), relatively stable value of oncology treatment, and are basically covered by national medical insurance; the determination of level I recommendations is not changed by commercial medical insurance, and the main consideration is clear benefit to patients.		
**Level II Recommendation**	**Types 1B evidence and some Types 2A evidence** In general, Type 1B evidence and some of the Type 2A evidence with a slightly lower level of expert consensus or less accessible in China are used as Level II recommendations. Specifically, level II recommendations have the following characteristics: high-level evidence from randomized controlled multicenter studies available internationally or nationally, but drugs or treatments with poor accessibility or low efficacy-price ratios that are beyond the affordability of the general population; for those measures that provide significant benefits but are expensive, they can also be considered as level II recommendations with oncologic therapeutic value as the main consideration.		
**Level III Recommendation#**	**Types 2B evidence and Types 3 evidence** For those diagnostic and therapeutic methods that are being explored and lack strong evidence-based medical evidence, but for which the expert group has unanimous consensus, they can be used as level III recommendations for the reference of doctors.		
**Not Recommended**	For those drugs or medical technologies for which there is sufficient evidence that they do not benefit patients, or even cause patient harm, and for which there is unanimous consensus in the expert group, it should be written “ not recommended “. This can be any category of evidence.		
Part 2 Type of Evidence			
Evidence Characteristics			The level of expert consensus
Type	Level	Source	
1A	High	Rigorous Meta-analysis, Large Randomized Controlled Clinical Study	Unanimous consensus (support ≥ 80%)
1B	High	Rigorous Meta-analysis, Large Randomized Controlled Clinical Study	Basically unanimous consensus, and with little controversy (60%-80% support)
2A	Slightly lower	Meta-analysis of general quality, small randomized controlled clinical studies, well-designed large retrospective studies, case-control studies	Unanimous consensus (support ≥ 80%)
2B	Slightly lower	Meta-analysis of general quality, small randomized controlled clinical studies, well-designed large retrospective studies, case-control studies	Basically unanimous consensus, and with little controversy (60%-80% support)
3	Low	Uncontrolled, single-arm clinical studies, case reports, expert opinion	No consensus and highly controversial (support <60%)

#CSCO AI does not contain Level III recommendations.

Two senior oncology researchers input the patient’s information into the CSCO AI system and generated artificial intelligence recommendations. Neither the researchers nor CSCO AI knew about the physician’s decision.

### 2.5 Guideline experts group and generation of guideline recommendations

The CSCO BC guideline experts group was comprised by 3 highly qualified physicians, two of whom were members of the CSCO BC experts committee that participated in the formulation and drafting of the CSCO BC guidelines. The experts group members reviewed the cases and made the relevant treatment recommendations independently by referring to the CSCO BC guidelines [Version 2021 (43–46)]. The recommendations that reached consensus of two or more experts were considered to be the final “Guideline Recommendations”. The treatment recommendations in the CSCO BC guidelines are divided into “Recommended” and “Not Recommended”, with “Recommended” further divided into “Level I Recommendation”, “Level II Recommendation” and “Level III Recommendation”. “Not Recommended” means that the patient is recommended to be exempted from the relevant treatment. The specific classification criteria for the level of recommendation are shown in **Table 1**.

### 2.6 Assessment indicators and their significance

The adjuvant treatment for early breast cancer includes adjuvant targeted therapy, adjuvant chemotherapy, adjuvant radiotherapy and adjuvant endocrine therapy; each stages is assessed independently and finally analyzed in aggregate. The following assessment indicators are included, as shown in **Figures 1A, B**.

#### 2.6.1 Concordance

**Figure 3:**
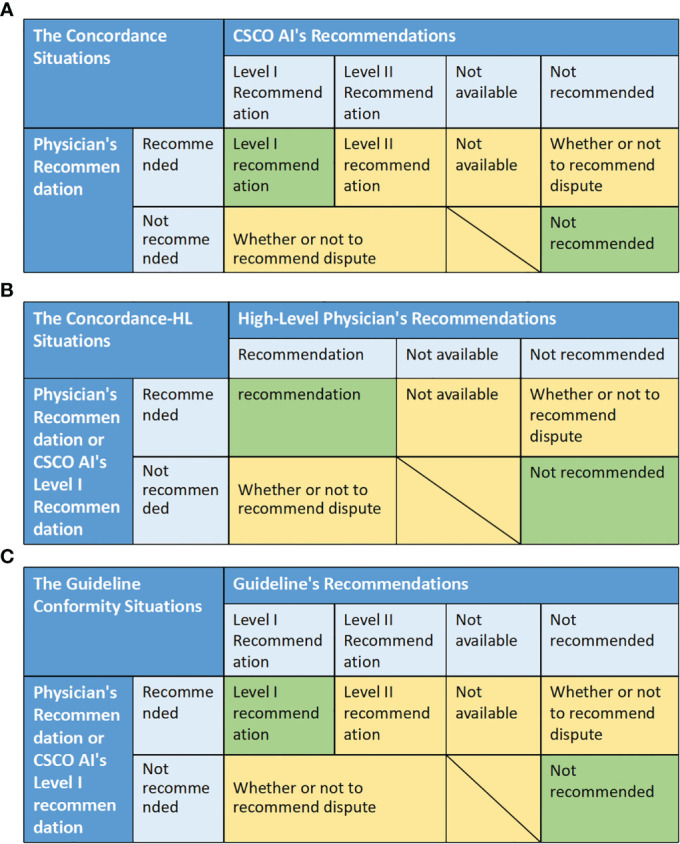
The matching situation between different decisions, and the evaluation criteria of related indicators. **(A)** The concordance situations between physician and CSCO AI recommendations. **(B)** The concordance situations between physician (or CSCO AI) and High-Level Physicians. **(C)** The guideline conformity situations between physician (or CSCO AI) and guideline.

Decision is considered as concordant when both the physician and the CSCO AI recommend the relevant treatment and the physician’s recommendation is consistent with the CSCO AI’s “Level I Recommendation” or neither is recommended, otherwise it is discordant. Specifically, decision concordance was assessed based on the match between physician and CSCO AI recommendations. There are five situations: “Level I recommendation” means that the physician’s recommendation is the same as the CSCO AI’s Level I recommendation; “Level II recommendation” means that the physician’s recommendation is the same as the CSCO AI’s Level II recommendation; “Not available” means that both the physician and CSCO AI recommended the relevant adjuvant therapy, but the physician’ recommendation was not considered by CSCO AI. “Not recommended” means that both the physician and the CSCO AI recommended not to receive the relevant adjuvant therapy. “ Whether or not to recommend dispute” means that the physician and CSCO AI dispute whether the patient should be recommended to receive the relevant adjuvant therapy. The “ Level I recommendation” and “ Not recommended” were considered to be concordance, while all other situations were discordance. Concordance = (number of concordance cases/total number of cases)*100%. (**Figure 3A**)

This index assesses the standardization treatment level of CDSS in view of empirical clinical level (i.e., by assessing the CDSS’ decision-making level is most similar to that of which level of physician, thus clarifying its clinical application value), and can also indirectly reflect the treatment differences across physicians.

#### 2.6.2 Concordance with high-level physicians

Decision is considered as concordant when both the physician (or CSCO AI’s “Level I Recommendation”) and the high-level physician recommend the relevant treatment and the specific program is same or neither is recommended, otherwise it is discordant. Specifically, decision concordance was assessed based on the match between physician (or CSCO AI’s “Level I recommendations’’) and high-level physician’ recommendations. There are four situations: “Recommended” means that both the physician (or CSCO AI) and the high-level physician recommend the relevant adjuvant therapy, and both the physician’s recommendation (or CSCO AI’s “Level I recommendation”) and the high-level physician’s specific recommendation are the same. “Not available” means that both the physician (or CSCO AI) and the high-level physician recommend the relevant adjuvant therapy, but the physician’ recommendation (or CSCO AI’s “Level I recommendation”) was not considered by high-level physician. “Not recommended” means that both the physician (or CSCO AI) and the high-level physician recommended not to receive the relevant adjuvant therapy. “ Whether or not to recommend dispute” means that the physician (or CSCO AI) and the high-level physician dispute whether the patient should be recommended to receive the relevant adjuvant therapy. The “ Recommendation” and “ Not recommended” were considered to be concordance, while all other situations were discordance. Concordance = (number of concordance cases/total number of cases)*100%. (**Figure 3B**)

This indicator takes high-level doctors (provincial senior physicians in this trial) as the standard for decision quality assessment, assesses the standardization treatment level of different physicians as and CDSS in view of the empirical clinical level, and can reflect the treatment differences across physicians.

#### 2.6.3 Consensus rate

Enrolled cases were randomly assigned to three differently senior physicians from different grades of hospitals in group format (G1-9), and each physician independently reviewed all cases in the case group. So, the group of decision-making physicians for the same case in this study consisted of three physicians of different seniority. For example, 59 cases in G1 were assigned to provincial junior physician 4 (3034), municipal senior physician 3 (2013) and county middle physician3 (1023) for decision-making at the same time; similarly, 59 cases in G9 were assigned to provincial senior physician 2 (3012), municipal middle physician 3 (2023) and county junior physician2 (1032) for decision-making at the same time. Consensus was considered to be reached when the decisions of the three physicians were in complete agreement; Otherwise, it was considered not reached. Consensus rate = (number of cases with consensus/total number of cases) * 100%.

This indicator assesses the treatment variation across physicians in view of the empirical clinical level, and a lower consensus rate reflects greater internal treatment variation.

#### 2.6.4 Decision stability

Decision stability was assessed based on changes in the discordance subgroup of physician and CSCO AI decisions. In the discordance subgroup, a decision is assessed as stable if the physician’s “Final Decision” is the same as the “Initial Decision”, otherwise it is assessed as decision instability. Decision stability = (number of cases with decision unchanged/number of all cases in the discordance subgroup with possible decision change) * 100%.

This indicator assesses the decision stability (or called anti-interference rate) of different physicians in view of the empirical clinical level, which is the probability of treatment strategy change when the physician is interfered by external opinions, and essentially reflects the maturity of the physician’s knowledge system; it can also reflect the internal variation of the physician’s technical maturity.

#### 2.6.5 Guideline conformity

It was considered as conforming to the guideline when both the physician (or CSCO AI’s “Level I Recommendations”) and the guideline recommended the relevant treatment and the physician’s recommendation was consistent with the guideline’s “Level I Recommendations” or neither was recommended, otherwise it was not conformity. Specifically, guideline conformity was assessed based on the match between physician and CSCO AI recommendations. There are five situations: “Level I recommendation” means that the physician’s recommendation (or CSCO AI’s Level I recommendations) is the same as guideline’s Level I recommendations; “Level II recommendation” means that the physician’s recommendation (or CSCO AI’s Level I recommendation) is the same as the guideline’s Level II recommendation; “Not available” means that both the physician (or CSCO AI) and guideline recommended the relevant adjuvant therapy, but the physician’ recommendation (or CSCO AI’ recommendation) was not considered by guideline. “Not recommended” means that both the physician (or CSCO AI) and the guideline recommended not to receive the relevant adjuvant therapy. “ Whether or not to recommend dispute” means that the physician (or CSCO AI) and guideline dispute whether the patient should be recommended to receive the relevant adjuvant therapy. The “ Level I recommendation” and “ Not recommended” were considered to be conformity, while all other situations were nonconformity. Conformity = (number of conformity cases/total number of cases)*100%. (**Figure 3C**)

The indicator assesses the standardization treatment level of CDSS and physicians at the level of pure guideline adherence, and can reflect internal treatment variation through inter-comparison.

#### 2.6.6 Calibration for concordance and guideline conformity

Select the physician subgroup with the maximum decision stability as the benchmark and calculate the relative index of decision stability for each physician subgroup. Relative index of decision stability = decision stability/maximum decision stability*100%. The index is used to calibrate the “Guideline Conformity” and “Concordance”. The calibrated indicator = relative index of decision stability * original indicator value.

By calibrating the concordance and guideline conformity with decision stability, we can get the calibrated concordance and the calibrated conformity that combine the technical maturity factors, and the calibrated indexes can more accurately reflect the treatment level of different doctors

### 2.7 Statistical analysis

Case characteristics included age, menstrual status, breast surgery modality, axillary surgery modality, TNM stage, molecular subtype and treatment stage; where qualitative data were expressed in the form of rates. Assessed indicators were expressed in the form of percentages. Univariate analysis was performed by chi-square test or McNemar test based on paired samples, and multivariate analysis was performed by logistic regression; differences were considered statistically significant when P<0.05.

Decision stability was compared between different seniority physician group of different grades of hospitals (101-303), and a propensity score matching analysis was performed on the “physician and CSCO AI decision discordance” data for each physician groups using a multivariate logistic regression model to achieve balancing and comparability at the baseline information level. Propensity score matching analysis was based on the following factors: Age, Menstrual Status, Breast Surgery Modality, Axillary Surgery Modality, TNM Stage, Molecular Subtype and Treatment Stage. Pairs of patients were derived using 1:1 greedy nearest neighbor matching within propensity score (PS) of 0.01. Using SPSS 26.0 software for data processing and analysis.

## 3 Results

### 3.1 Clinical characteristics of cases

531 breast cancer patients were enrolled in this study. Patients were randomly assigned to nine groups (G1-9) for review by different physicians; **Table 2** shows the clinical characteristics of the different case groups, which were balanced and comparable in terms of baseline indicators (*P*>0.05). As shown in **Table S1**, in the physician and CSCO AI decision discordance subgroups, propensity score-matched analysis resulted in 55 matched pairs in each group and each case group was balanced and comparable at the baseline information level.

**Table 2:** Clinical Characteristics of Different Case Groups.

Characteristic	Total	Group 1 (N=531)	Group 2 (N=59)	Group 3 (N=59)	Group 4 (N=59)	Group 5 (N=59)	Group 6 (N=59)	Group 7 (N=59)	Group 8 (N=59)	Group 9 (N=59)	*χ* ^2^	*P*
**Female, %**	531 (100.0)	59 (100.0)	59 (100.0)	59 (100.0)	59 (100.0)	59 (100.0)	59 (100.0)	59 (100.0)	59 (100.0)	59 (100.0)		
Age, n (%)												
≤44	143 (26.9)	11 (18.6)	13 (22.0)	18 (30.5)	17 (28.8)	14 (23.7)	15 (25.4)	17 (28.8)	21 (35.6)	17 (28.8)	13.303	0.65
45–54	255 (48.0)	33 (55.9)	31 (52.5)	25 (42.4)	24 (40.7)	32 (54.2)	27 (45.8)	30 (50.8)	21 (35.6)	32 (54.2)		
≥55	133 (25.0)	15 (25.4)	15 (25.4)	16 (27.1)	18 (30.5)	13 (22.0)	17 (28.8)	12 (20.3)	17 (28.8)	10 (16.9)		
Menstrual status, n (%)												
Premenopausal	319 (60.1)	33 (55.9)	35 (59.3)	36 (61.0)	31 (52.5)	35 (59.3)	41 (69.5)	35 (59.3)	37 (62.7)	36 (61.0)	4.256	0.833
Postmenopausal	212 (39.9)	26 (44.1)	24 (40.7)	23 (39.0)	28 (47.5)	24 (40.7)	18 (30.5)	24 (40.7)	22 (37.3)	23 (39.0)		
Breast Surgery Modality, n (%)												
BCS	96 (18.1)	12 (20.3)	8 (13.6)	13 (22.0)	9 (15.3)	11 (18.6)	6 (10.2)	13 (22.0)	13 (22.0)	11 (18.6)	5.722	0.678
SM	435 (81.9)	47 (79.7)	51 (86.4)	46 (78.0)	50 (84.7)	48 (81.4)	53 (89.8)	46 (78.0)	46 (78.0)	48 (81.4)		
Axillary surgery modality, n (%)												
ALND	379 (71.4)	37 (62.7)	39 (66.1)	43 (72.9)	47 (79.7)	40 (67.8)	40 (67.8)	45 (76.3)	45 (76.3)	43 (72.9)	11.041	0.807
SLNB	152 (28.6)	22 (37.3)	20 (33.9)	16 (27.1)	12 (20.3)	19 (32.2)	19 (32.2)	14 (23.7)	14 (23.7)	16 (27.1)		
TNM Stage, n (%)												
I	191 (36.0)	27 (45.8)	20 (33.9)	23 (39.0)	23 (39.0)	16 (27.1)	25 (42.4)	19 (32.2)	19 (32.2)	19 (32.2)	7.208	0.514
II	265 (49.9)	25 (42.4)	30 (50.8)	26 (44.1)	27 (45.8)	36 (61.0)	29 (49.2)	35 (59.3)	33 (55.9)	24 (40.7)		
III	75 (14.1)	7 (11.9)	9 (15.3)	10 (16.9)	9 (15.3)	7 (11.9)	5 (8.5)	5 (8.5)	7 (11.9)	16 (27.1)		
Molecular subtype, n (%)												
HER2 positive HR negative	48 (9.0)	2 (3.4)	6 (10.2)	5 (8.5)	2 (3.4)	11 (18.6)	4 (6.8)	7 (11.9)	7 (11.9)	4 (6.8)	40.52	0.144
HER2 positive HR positive	111 (20.9)	15 (25.4)	13 (22.0)	11 (18.6)	8 (13.6)	9 (15.3)	18 (30.5)	9 (15.3)	14 (23.7)	14 (23.7)		
Luminal A	81 (15.3)	9 (15.3)	7 (11.9)	10 (16.9)	10 (16.9)	10 (16.9)	6 (10.2)	5 (8.5)	8 (13.6)	16 (27.1)		
Luminal B (HER2 negative)	223 (42.0)	24 (40.7)	24 (40.7)	26 (44.1)	34 (57.6)	22 (37.3)	20 (33.9)	32 (54.2)	22 (37.3)	19 (32.2)		
TNBC	68 (12.8)	9 (15.3)	9 (15.3)	7 (11.9)	5 (8.5)	7 (11.9)	11 (18.6)	6 (10.2)	8 (13.6)	6 (10.2)		

P values are from Chi-square test of differences across different Case Groups.

HER2=human epidermal growth factor receptor 2. HR=hormone receptor. Luminal A=HER2 negative HR positive, and PR≥20% and Ki-67 <15%. Luminal B (HER2 negative) =HER2 negative HR positive, and PR < 20% or Ki-67≥15%. TNBC=triple-negative breast cancer (HR and HER2 negative-tumors). PR=progesterone receptor. Ki-67 = proliferating cell nuclear antigen-67. BCS=breast conserving surgery. SM=simple mastectomy. SLNB=sentinel lymph node biopsy. ALND=axillary lymph node dissection.

### 3.2 Decision concordance between physicians and CSCO AI

**Figure 4:**
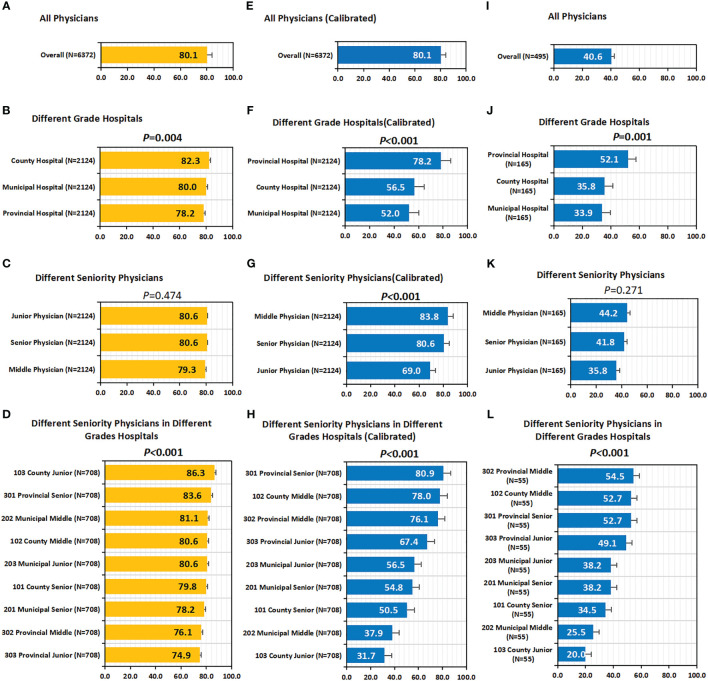
Concordance, calibrated concordance and decision stability. **(A–D)** Concordance. **(A)** Concordance of all Physicians; **(B)** Concordance of Different Grade Hospitals; **(C)** Concordance of Different Seniority Physicians; **(D)** Concordance of Different Seniority Physicians in Different Grades Hospitals. **(E–H)** Calibrated Concordance. **(E)** Calibrated Concordance of all Physicians; **(F)** Calibrated Concordance of Different Grade Hospitals; **(G)** Calibrated Concordance of Different Seniority Physicians; **(H)** Calibrated Concordance of Different Seniority Physicians in Different Grades Hospitals. **(I–L)** Decision Stability. **(I)** Decision Stability of all Physicians; **(J)** Decision Stability of Different Grade Hospitals; **(K)** Decision Stability of Different Seniority Physicians; **(L)** Decision Stability of Different Seniority Physicians in Different Grades Hospitals. P values are from Chi-square test of differences across different Case Groups.

Overall, the concordance between CSCO AI and physicians’ decisions was 80.1% (5107/6372) (**Figures 4A, E**); Subgroup analysis showed that the decision concordance was influenced by physicians’ seniority and hospitals’ grade (**Figures 4B-D, F–H**).

Overall, the decision concordance with the CSCO AI differed by physician’ seniority; before calibration, no statistically significant differences were found between different seniority physicians (80.6% vs. 79.3% *vs*. 80.6%, *P*=0.474); after calibration, it was observed that senior and middle physicians had significantly higher calibrated concordance than junior physicians (80.6%, 83.8% *vs.* 69.0%; *P<*0.001). Overall, the decision concordance with the CSCO AI differed across different grades of hospitals; in particular, before calibration, county hospitals had the highest concordance compared to municipal and provincial hospitals (82.3% *vs*. 80.0% and 78.2%; *P*=0.004); after calibration, it is observed that provincial hospital had significantly higher calibrated concordance than county and municipal hospital physicians (78.2% *vs.* 56.5% and 52.0%; *P<*0.001). Overall, the decision concordance with the CSCO AI differed across different seniority physicians in different grades of hospitals; before calibration, provincial-senior physicians (301) and county-junior physicians (103) had a higher concordance than the other physicians, but no statistical difference was found between them (83.6% *vs.* 86.3%, *P*=0.052); after calibration, it was observed that provincial-senior physicians (301) had a significantly higher calibrated concordance than other physicians (*P*<0.001). The concordance between different physicians and CSCO AI fluctuated between 62.7% and 93.2% (*P*<0.001) (**Table S2**).

Additional details about the univariate analyses performed by the clinical characteristics of the cases are available in **Table S2**. Logistic regression analysis showed that decision-making physician, breast surgery modality, TNM stage, molecular subtype, and treatment stage were independent risk factors of concordance (*P*<0.05); whereas patient age, menstrual status, and axillary surgery modality had no effect on concordance (*P*>0.05) (**Table S3**).

### 3.3 Reasons and cases of decision discordance between CSCO AI and physicians

As shown in **Table 3**, overall, there were 1265 discordance cases, of which 68.1% (862/1265) were caused by treatment strategy differences, 10.0% (126/1265) were caused by physicians’ misreading or omission of important disease information, and another 21.9% (277/1265) of cases were caused by physicians’ errors in assessing concordance between themselves and CSCO AI’ decisions. There was a statistical difference (*P*<0.05) in the reasons of discordance between different physicians and CSCO AI decisions. Specific discordance cases are described in **Table S4**.

**Table 3:** Reasons for Discordance between Physician and CDSS Decisions.

Variable	N	Treatment strategy differences, n (%)	Missing or misreading information, n (%)	Incorrect concordance assessment, n (%)	*χ* ^2^	*P*
**Overall**	1265	862 (68.1)	126 (10.0)	277 (21.9)		
Different Seniority Physicians					13.888	0.008
Senior Physician	413	296 (71.7)	36 (8.7)	81 (19.6)		
Middle Physician	440	311 (70.7)	47 (10.7)	82 (18.6)		
Junior Physician	412	255 (61.9)	43 (10.4)	114 (27.7)		
Different Grade Hospitals					12.831	0.012
Provincial Hospital	463	299 (64.6)	61 (13.2)	103 (22.2)		
Municipal Hospital	425	311 (73.2)	29 (6.8)	85 (20.0)		
County Hospital	377	252 (66.8)	36 (9.5)	89 (23.6)		
Different Seniority Physicians in Different Grades Hospitals					113.166	< 0.001
301 Provincial Senior	116	88 (75.9)	18 (15.5)	10 (8.6)		
302 Provincial Middle	169	120 (71.0)	21 (12.4)	28 (16.6)		
303 Provincial Junior	178	91 (51.1)	22 (12.4)	65 (36.5)		
201 Municipal Senior	154	104 (67.5)	11 (7.1)	39 (25.3)		
202 Municipal Middle	134	121 (90.3)	7 (5.2)	6 (4.5)		
203 Municipal Junior	137	86 (62.8)	11 (8.0)	40 (29.2)		
101 County Senior	143	104 (72.7)	7 (4.9)	32 (22.4)		
102 County Middle	137	70 (51.1)	19 (13.9)	48 (35.0)		
103 County Junior	97	78 (80.4)	10 (10.3)	9 (9.3)		

P values are from Chi-square test.

### 3.4 Decision concordance with High-Level physicians

**Figure 5:**
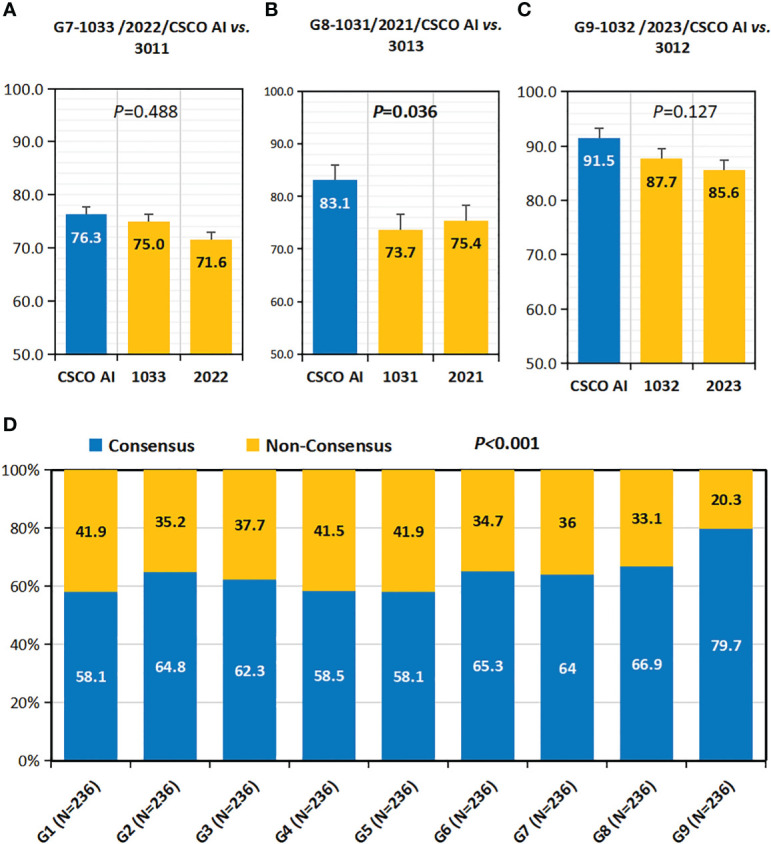
Concordance with high-level physicians and consensus rate. **(A–C)** Concordance with High-Level Physicians(Concordance-HL). **(A)** Concordance-HL of 1033,2022 and CSCO AI; **(B)** Concordance-HL of 1031,2021 and CSCO AI; **(C)** Concordance-HL of 1032,2023 and CSCO AI. **(D)** Consensus Rate. P values are from Chi-square test of differences across different Case Groups.

As shown in **Figures 5A–C**, in all case groups (G7,G8 and G9), the CSCO AI had higher “decision concordance with high-level physicians” than all decision physicians (G7:76.3% *vs.* 75.0%, 71.6%, *P*=0.488; G8:83.1% *vs.* 73.7%, 75.4%, *P*=0.036; G9:91.5% *vs.* 87.7%, 85.6%, *P*=0.127).

Additional details about the univariate analyses performed by the clinical characteristics of the cases are available in **Table S5**. Logistic regression analysis showed that decision-making physician, patient’ age, menstrual status, breast surgery modality, TNM stage, molecular subtype, and treatment stage were independent risk factors of concordance-HL (*P*<0.05); whereas axillary surgery modality had no effect on concordance-HL (*P*>0.05) (**Table S6**).

### 3.5 Consensus rate

Overall, the physicians’ consensus rate was 64.2% (1363/2124) (**Figure 5D**). there was variability in the consensus rate among the nine physician groups (G1-9), with G9 having the highest rate (79.7%).

Additional details about the univariate analyses performed by the clinical characteristics of the cases are available in **Table S7**. Logistic regression analysis showed that decision-making physician, breast surgery modality, molecular subtype, and treatment stages were independent risk factors of consensus rate (*P*<0.05) (**Table S7**).

### 3.6 Decision stability

Overall, physicians’ decision stability was 40.6% (201/495) (**Figure 4I**). Subgroup analysis showed that decision stability was influenced by physicians’ seniority and hospitals’ grade (**Figures 4J–L**). Senior and Middle physicians were slightly higher than junior physicians (41.8%, 44.2% *vs.* 35.8%; *P*=0.271). Provincial hospitals had significantly higher decision stability than county and municipal hospitals (52.1% *vs.* 35.8% and 33.9%; *P*=0.001). Overall, decision stability differed across different seniority physicians in different grade hospitals; compared to other physicians, provincial-middle physicians (302, 54.5%) were the highest, followed by provincial-senior physicians (301, 52.7%) and county-middle physicians (102, 52.7%).

Additional details about the univariate analyses performed by the clinical characteristics of the cases are available in **Table S8**. Logistic regression analysis showed that decision-making physician, menstrual status, and treatment stage were independent risk factors of decision stability (*P*<0.05) (**Table S8**).

### 3.7 Guideline conformity of physician’ decision-making

**Figure 6:**
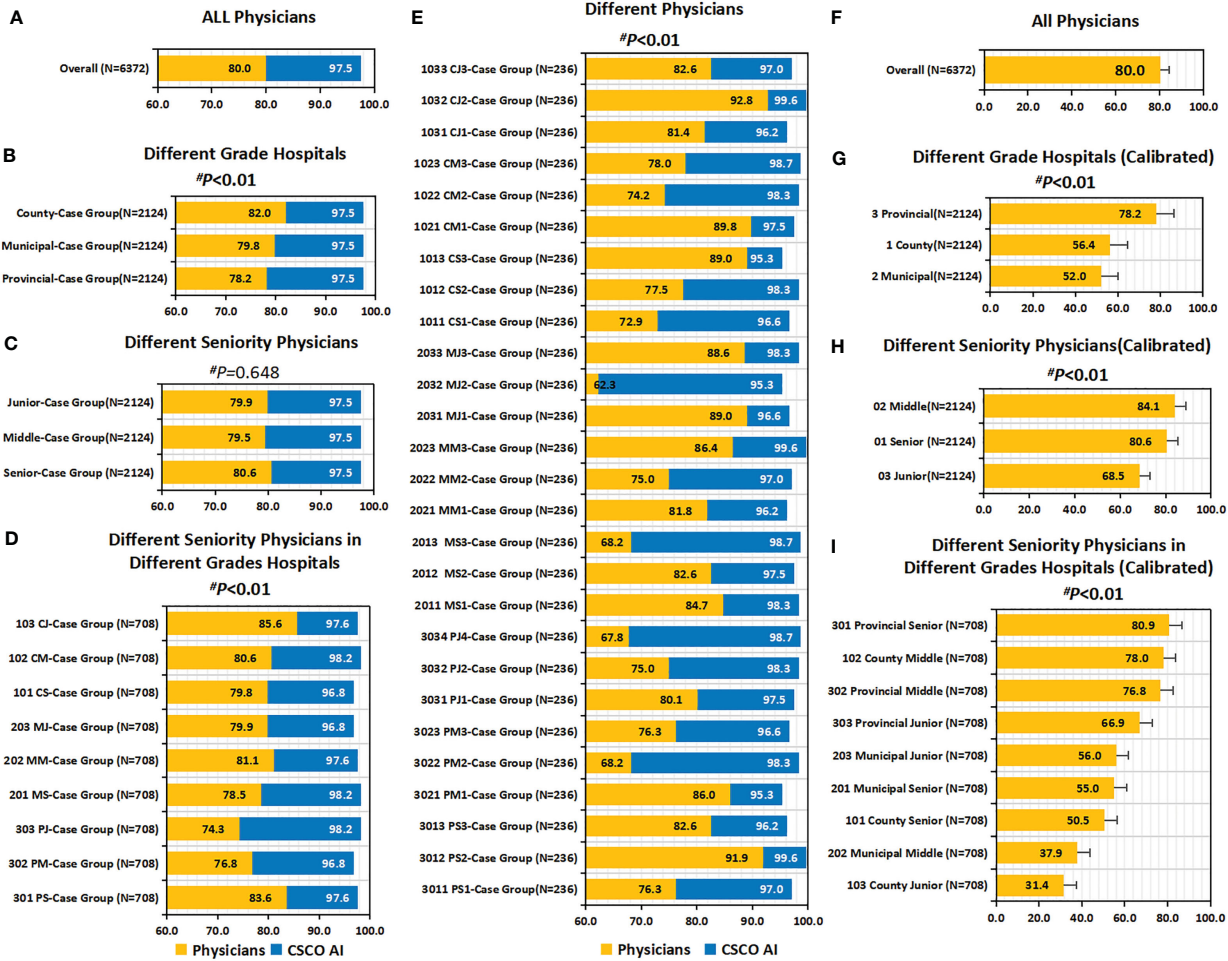
Guideline conformity and calibrated guideline conformity. **(A–E)** Guideline Conformity. **(A)** Guideline Conformity of All Physicians; **(B)** Guideline Conformity between Different Grade Hospitals; **(C)** Guideline Conformity between Different Seniority Physicians; **(D)** Guideline Conformity between Different Seniority Physicians in Different Grades Hospitals; **(E)** Guideline Conformity between different independent physicians. **(F–I)** Calibrated Guideline Conformity. **(F)** Calibrated Guideline Conformity between different independent physicians; **(G)** Calibrated Guideline Conformity between Different Grade Hospitals; **(H)** Calibrated Guideline Conformity between Different Seniority Physicians; **(I)** Calibrated Guideline Conformity between Different Seniority Physicians in Different Grades Hospitals. The comparison of the difference in guideline conformity between paired samples of physicians and CDSS was performed using the McNemar test, and all p values were less than 0.01. P values are from Chi-square test of physicians’ guideline conformity differences. PS=Provincial Senior Physicians. PM=Provincial Middle Physicians. PJ=Provincial Junior Physicians. MS=Municipal Senior Physicians. MM=Municipal Middle Physicians. MJ=Municipal Junior Physicians. CS=County Senior Physicians. CM=County Middle Physicians. CJ=County Junior Physicians. CG=Case Groups. CSCO AI= the Chinese Society of Clinical Oncology Artificial Intelligence System.

Overall, the guideline conformity of physician’ decision-making was 80.0% (5100/6372) (**Figures 6A, F**). Subgroup analysis showed that the conformity was influenced by physician’ seniority and hospital’ level (**Figures 6B–E**, **G–I**). Overall, conformity differed across physicians’ seniority; before calibration, senior physicians had a slightly higher conformity than middle and junior physicians, but the difference was not statistically significant (80.6% *vs.* 79.5%, 79.9%; *P*=0.648); after calibration, it was observed that senior and middle physicians had significantly higher calibrated guideline conformity than junior physicians (80.6%, 84.1% *vs.* 68.5%; P<0.001). Overall, the guideline conformity varied across different grades of hospitals; in particular, before calibration, county hospitals had the highest conformity compared to municipal and provincial hospitals (82.0% *vs.* 79.8% and 78.2%; *P*=0.009); after calibration, it was observed that provincial hospitals had significantly higher calibrated guideline conformity than county and municipal hospitals (78.2% *vs.* 56.4% and 52.0%; *P*<0.001). Overall, guideline conformity differed across different seniority physicians in different grades of hospitals; before calibration, provincial-senior physicians (301) and county-junior physicians (103) had higher conformity than other physicians, but no statistical difference was seen between them (83.6% *vs.* 85.6%, *P*=0.303); after calibration, it was found that provincial-senior physicians (301) had significantly higher calibrated guideline conformity than other physicians (*P*<0.001). The conformity fluctuated between 62.3% and 92.8% across physicians (P<0.001).

In addition, a univariate sublevel analysis for overall conformity showed that physician’ guideline conformity was also influenced by patient’ age, menstrual status, breast surgery modality, axillary surgery modality, TNM stage, molecular subtype, and treatment stage **Table S9**). Before calibration, guideline conformity was highest in patients aged ≥55 years across all age groups (82.0% *vs.* 80.2% and 78.9%; P=0.039); but after calibration, however, guideline conformity of patients aged ≥55 years was lowest (58.7% *vs.* 70.5% and 78.9%; *P*<0.001). Postmenopausal patients had a slightly higher guideline conformity rate than premenopausal patients before calibration, but no statistical significance (81.0% *vs.* 79.4%; P=0.127); after calibration it was shown that postmenopausal patients had a significantly lower guideline conformity rate than premenopausal patients (57.1% *vs.* 79.4%; *P*<0.001). For patients with different breast surgery modalities, the guideline conformity of simple mastectomy (SM) was significantly higher than breast conserving surgery (BCS) (before calibration: 81.6% *vs.* 73.2%, *P*<0·001; after calibration: 81.6% *vs.* 69.8%, *P*<0.001). For patients with different axillary surgical modalities, guideline conformity of ALND was higher than SLNB (before calibration: 81.2% *vs.* 77.1%, *P*<0.001; after calibration: 81.2% *vs.* 75.8%, *P*<0.001). For patients with different stages, the conformity of Stage III patients was significantly higher than that of Stage I and II patients (before calibration: 83.4% *vs.* 78.3% and 80.3%, P=0.004; after calibration: 83.4% *vs.* 68.5% and 60.2%, *P*<0.001). Before calibration, the guideline conformity of TNBC was significantly higher than other breast cancer subtypes (87.7% *vs.* 79.0%, 74.1%, 83.1% and 79.7%; *P*<0.001); however, after calibration, the Luminal type had higher guideline conformity than other subtypes, with the highest rate in Luminal A (85.9%) and followed by the Luminal B (HER2 negative) type (79.7%). Before correction, the adjuvant targeted therapy stage had the highest guideline conformity rate across all adjuvant treatment stages (89.8% *vs.* 67.5%, 80.3% and 82.5%; *P*<0.001); however, after correction, the adjuvant endocrine therapy stage had the highest guideline conformity rate (82.5%), while the adjuvant targeted therapy stage had the lowest (49.7%), with a statistically significant difference (*P*<0.001).

Logistic regression analysis showed that decision-making physician, breast surgery modality, TNM stage, molecular subtype, and treatment stage were independent risk factors of guideline conformity (*P*<0.05); whereas patient’ age, menstrual status, and axillary surgery modality had no effect on guideline conformity (*P*>0.05)(**Table S10**).

### 3.8 Guideline conformity of CSCO AI’ decision-making

Overall, the guideline conformity of CSCO AI’ decision-making was 97.5% (6213/6372) (**Figure 6A**). In addition, a univariate sublevel analysis for overall conformity showed that guideline conformity of CSCO AI was influenced by patient’ age, TNM stage, molecular subtype, and treatment stage (**Table S9**). Overall, guideline conformity in patients ≥55 years old was the highest across all age stages (98.3% *vs.* 97.7% and 97.0%; *P*=0.016); guideline conformity differed by TNM stage, with stage III patients having a higher conformity rate than stage I and II patients (99.7% *vs.* 96.9% and 97.4%; *P*<0.001); conformity differed by molecular subtypes, with TNBC having the highest conformity rate (99.3% *vs.* 94.8%, 96.6%, 96.3% and 98.4%; *P*<0.001); and the performance of CSCO AI in these subgroups was similar to that of overall physicians. Overall, guideline conformity varied by treatment phase, with the adjuvant radiotherapy phase having the highest conformity (99.8%), while adjuvant chemotherapy had the lowest conformity (94.9%) and adjuvant targeted and adjuvant endocrine therapy had moderate conformity (98.1% and 97.2%, respectively), with statistically significant differences (*P*<0.001); and the performance of CSCO AI in these subgroups differed from that of overall physicians. No statistical differences were seen in guideline conformity between different physician case groups, different menstrual status, breast surgery modalities and axillary surgery modalities (*P*>0.05).

Logistic regression analysis showed that patient’ age, breast surgery modality, axillary surgery modality, TNM stage, molecular subtype, and treatment stage were independent risk factors of guideline conformity (*P*<0.05); while different decision-making physician case groups and patient’ menstrual status had no effect on guideline conformity (*P*>0.05)(**Table S10**).

### 3.9 Comparison of guideline conformity between CSCO AI and physicians

Overall, CSCO AI had a significantly higher guideline conformity than physicians (97.5% *vs.* 80.0%; *P*<0.001), with a 17.5% conformity difference (**Figure 6A**). Subgroup analyses performed for different physicians showed that CSCO AI had a significantly higher guideline compliance than the performance of all decision-making physicians, with a statistically significant difference (*P*<0.01) (**Figures 6B–E**). And the guideline compliance rate of CSCO AI was significantly higher than physicians in different subgroups of patient’ age, menstrual status, breast surgery modality, axillary surgery modality, TNM stage, molecular subtype and treatment stage (*P*<0.01) (**Table S9**).

### 3.10 Comparison of internal variation in guideline conformity between CSCO AI and physicians

**Figure 7:**
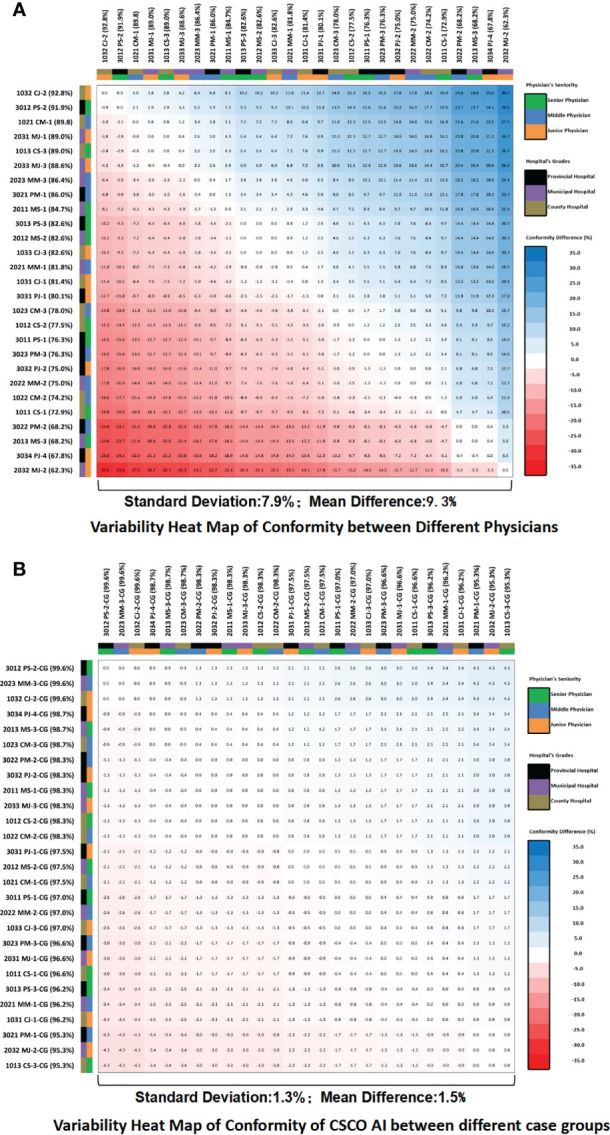
Guideline conformity variability heat map. **(A)** Variability heat map of guideline conformity between different physicians; **(B)** Variability heat map of guideline conformity of CSCO AI between different case groups. The values in the heat map squares represent the differences in the guideline conformity rates between the horizontal and vertical subgroups corresponding to each square. If the difference is negative, the color of the square is red; if the difference is positive, the color of the square is blue; and the larger the absolute value of the difference, the darker the color of the square. PS, Provincial Senior Physicians; PM, Provincial Middle Physicians; PJ, Provincial Junior Physicians; MS, Municipal Senior Physicians; MM, Municipal; Middle Physicians; MJ, Municipal Junior Physicians; CS, County Senior Physicians; CM, County Middle Physicians; CJ, County Junior Physicians; CG, Case Groups; CSCO AI, the Chinese Society of Clinical Oncology Artificial Intelligence System.

The standard deviation of guideline conformity among different physicians was 7.9%, with a mean difference of 9.3% (**Figure 7A**). the standard deviation of guideline conformity of CSCO AI among different physician case groups was 1.3%, with a mean difference of 1.5% (**Figure 7B**). Compared to physicians, CSCO AI had less internal variation in decision-making, with a difference of 6.6% in the standard deviation of guideline conformity and a difference of 7.8% in the mean difference.

### 3.11 Reasons for CSCO AI non-conformity with guidelines and specific cases

Nonconformity occurred in 2.5% (159/6372) of the cases overall in the decision-making performed by CSCO AI. The percentage of nonconformity that occurred varied by treatment stage, with the adjuvant chemotherapy stage having the highest rate of 5.1% (81/1593), and the adjuvant radiotherapy stage having the lowest rate of 0.2% (3/1593); in addition, the adjuvant targeted therapy stage had 1.9% (30/1593), and the adjuvant endocrine therapy stage had 2.8% (45/1593). Except for adjuvant radiotherapy decision nonconformity, which was due to misidentification of case information, all other nonconformities with the guidelines were due to treatment strategy differences.

**Table S11** describes the specific cases and reasons for nonconformity with the guidelines in different treatment stages.

## 4 Discussion

How is the current status of standardized treatment variation among different geographical regions and how can it be comprehensively assessed? How is the potential for clinical application of CDSS in different geographical regions and how can it be assessed? And How can the actual clinical impact of CDSS be comprehensively evaluated, especially in enhancing the level of physicians’ standardized decision-making and balancing the variation of standardized treatment among different geographical regions? The resolution of these questions is extremely important for advancing the rational clinical application of CDSS and improving the current dilemma of oncology treatment.

We have found that the existing indicators of “ decision concordance” and “guideline conformity” (23–38) have significant limitations in assessing the standardization treatment level of physicians and CDSS. In this trial we observed a noteworthy phenomenon, in which the decision concordance between the county-junior physicians (103) and CDSS was the highest among all physicians (86.3%), which seems to indicate that the decision quality of CDSS is similar to that of county-junior physicians, which is at a low level. Similarly, Xu F et al. (33) showed that the decision concordance of WFOs and junior physicians was higher than that of senior and middle physicians in their study (0.68 vs. 0.54 and 0.49, P=0.001). As well, we also observed that many junior physicians in this trial had higher guideline conformity rates than middle and senior physicians, and the most notable was the county junior physicians, who had the highest guideline conformity rate (85.6%) among all physicians, which was even slightly higher than the provincial senior physicians (85.6% vs. 83.6%), and this phenomenon is inconsistent with the clinical reality and our perception. In our opinion, this is because existing indicators of “Decision Concordance” and “Guideline Conformity” do not take into account the impact of physicians’ expertise maturity on their decision-making performance. The junior physicians usually have a higher level of adherence to their senior physicians and guidelines at the beginning of their careers because they do not yet have the same level of clinical experience as the senior physicians, and are easily influenced by external opinions to make changes in their decisions.

In this context, we have introduced the indicator “Decision Stability” for the first time as an empirical indicator to assess the expertise maturity of CDSS and physicians. This indicator assesses the stability of different physicians’ decisions in terms of their experience clinical level, and is the probability of changes in treatment strategies when physicians are disturbed by external opinions, essentially reflecting the maturity of physicians’ expertise system. During the analysis of decision stability, we observed that county junior physicians had the lowest decision stability, and provincial middle senior physicians (302, 54.5%) had the highest, followed by provincial senior physicians (301, 52.7%), which is consistent with our expectations. Therefore, we calibrated the “Decision Concordance” and “Guideline Conformity” indicators using “Decision Stability” so as to correct the negligence of these indicators on the technical maturity, and developed the indicators named “Calibrated Decision Concordance” and “Calibrated Guideline Conformity”, respectively. After being Calibrated by “decision stability”, we observed that CDSS and provincial senior physicians (301) had the highest decision concordance (80.9%), while that of CDSS and county junior physicians (103) was the lowest (31.7%). Similarly, after being corrected by “Decision Stability”, we observed that provincial senior physicians (80.9%) had a significantly higher “ calibrated guideline conformity rate” than other strata, while county junior physicians had the lowest (31.4%). That suggests that we should accurately understand the correct meaning of existing indicators like “Decision Concordance” and “Guideline Conformity”. The “Decision Concordance” only reflects the same situation between CDSS and physician decisions, it does not reflect whether the decisions are standardized and individualized to patients. The level of decision concordance does not directly indicate the standardization treatment level of CDSS and physicians. The value of the study of decision concordance is that it can reflect the treatment variation among different physicians as well as empirically assess the decision level of CDSS and initially evaluate whether it has the potential application value in clinical practice. And the “Corrected Concordance” corrected by “Decision Stability” showed better ability in reflecting the standardization level of physicians’ decisions. Similarly, “Guideline Conformity” only reflects the simple conformity between physician’s decision and guideline recommendation in a certain cross-section, and which is accurate in simply assessing the standardization level of physician’s decision, but cannot fully reflect the physician’s treatment level, and needs to consider the factor of technical maturity. The “ Calibrated Guideline Conformity” Calibrated by “Decision Stability” shows a better comprehensive evaluation ability in reflecting the standardization level of physicians and CDSS decisions, and the evaluation results are more in line with the actual clinical situation, because it takes into account the pure standardization based on “Guideline Conformity” and the empirical factor of technical maturity based on “Decision Stability”.

Additionally, it is worth noting that indicators such as “Decision Concordance” and “Guideline Conformity” are strongly influenced by the evaluation standards, and there are still no standard reporting principles, which largely affects the comparability between different studies. In the previous clinical studies related to WFO, “ Recommended” (equivalent to “Level I recommendation” and “Not recommended” in this study) and “ For consideration” (equivalent to “Level II recommendation” in this study) were classified as concordant (23–38). In this study, we adopted more stringent assessment criteria and classified “Level II recommendation” as discordant. Because the “ Level I recommendation” is a universal treatment choice with strong evidence and good accessibility, relatively stable tumor treatment value, and clear patient benefit, it is the first choice to be considered in clinical practice.

In addition, besides the standardization treatment level, internal decision variation, especially among different medical regions, is also worth considering. Therefore, we also suggested using “ Concordance Variation”, “Consensus Rate”, and “Guideline Conformity Variation” to assess the intra-physician decision variation, in which “Guideline Conformity Variation” can also be used to assess the decision variation of CDSS among different case groups, which also reflects the decision stability of CDSS. While “ Concordance with High-Level Physicians’ decision making” used high-level physicians (provincial senior physicians in this trial) as a criterion to evaluate the decision quality, it assessed the standardized treatment level of different physicians and CDSS in terms of clinical experience and could reflect the treatment variation among physicians. In summary, this study constructed a multi-level, multi-indicator system to comprehensively assess the standardized treatment level of physicians and CDSS, considering the level of empirical clinical and guidelines adherence-based purely standardization level as well as the comprehensive level.

Based on this indicator system we first assessed the standardized treatment level and the current status of internal variation among different seniority physicians in different grades of hospitals. At the level of empirical clinical data, different physicians’ decision concordance with CDSS and concordance with high-level physicians were significantly different, fluctuating between 62.7%-93.2% and 71.6%-87.7%, respectively; the overall consensus rate among physicians was 64.2%; the decision stability varied among physicians, compared with other physicians, provincial-middle physicians (54.5%) had the highest, followed by provincial-senior physicians and county-middle physicians (both 52.7%). In the purely standardization level, guideline conformity differed among physicians, with provincial-senior physicians (83.6%) and county-junior physicians (85.6%) having higher conformity than other physicians, but no statistical difference was seen between them; the standard deviation of guideline conformity among physicians was 7.9%, and the mean difference of 9.3%; after calibration, it was observed that at the comprehensive level provincial-senior physicians had significantly higher calibrated guideline conformity than other physicians. The study showed that there is significant internal variation in the standardization treatment levels of different seniority physicians in different levels of hospitals.

At the same time, the standardization treatment level of CDSS and its clinical application potential were further comprehensively assessed by comparing it with physicians. At the empirical clinical level, the overall decision concordance between the CDSS and physicians was 80.1%, and the calibrated concordance with provincial-senior physicians was significantly higher than other physicians; at the same time, the CDSS had a higher “decision concordance with high-level physicians” than all physicians, which fluctuated between 76.3%-91.5%. At the purely standardization level, CDSS had significantly higher guideline conformity than the performance of all physicians; the overall difference of conformity reached 17.5% (97.5% *vs.* 80.0%; *P*<0.001); the standard deviation of guideline conformity of CDSS among different physician case groups was 1.3%, and the mean difference was 1.5%; CDSS had less internal variation in treatment decision-making compared with physicians, and the difference of standard deviation of guideline conformity was 6.6%, and the difference of mean difference was 7.8%. Studies have shown that compared to physicians, CDSS have higher treatment standardization and less internal variation.

The study results show that CDSS, represented by CSCO AI, has significant potential to become an important component of the future healthcare system (47). First, it can help physicians identify and correct obvious errors timely during their heavy workload (48). In discordance cases, 10.0% of the discordances were caused by physicians’ misreading or omission of important medical information; for example, in Case three (pT1cN1M0 Stage IIA LuminalA), the physician had omitted the important medical information of N1 and did not recommend the patient to receive chemotherapy in the initial decision, and after being prompted by CDSS, the physician reviewed the case and realized the problem, and finally adopted anthracycline combined with cyclophosphamide (AC) program consistent with the CDSS level I recommendations (**Table S4**). Secondly, it can promote physicians’ standardized treatment by providing immediate and standardized reference opinions for all levels physicians in the practice, which is especially helpful for middle and junior physicians in municipal and county hospitals. Third, it helps physicians to improve their knowledge and decision-making skills constantly while working; CSCO AI provides relevant clinical guideline basis, reference literature and other information for all recommended treatment suggestions, which can promote physicians’ thinking and re-examination of relevant treatment programs and re-learning of relevant evidence-based medical evidence, especially when physicians and CDSS make discordant decisions.

Overall, discordance between CDSS and physician decision-making occurred in 19.9% of cases. Discordance was mainly caused by treatment strategy differences (68.1%, 862/1265), and was concentrated among stage I and II patients (92.5%, 797/862). In fact, whether pT1N0M0 requires chemotherapy has been a controversial clinical issue (49–51); for example, discordance case one is a T1cN0M0 (Luminal B (HER2-) type) in which the decision physician recommended AC (Adriamycin/cyclophosphamide) regimen chemotherapy for 4 cycles or consideration of further polygenic testing for evaluation, but CDSS did not recommend chemotherapy (**Table S4**). The remaining 10.0% discordance cases were due to physician misreading or missing important disease information. A further 21.9% discordance cases were due to physician errors in assessing the concordance between their “Initial Decision” and the “CDSS Recommendation”.

Overall, a total of 2.5% (159/6372) nonconformity cases occurred in the decision-making performed by the CDSS. It was mainly caused by treatment strategy differences, and was mainly concentrated in adjuvant chemotherapy and adjuvant endocrine therapy. For example, nonconformity case one is a T1aN0M0 (Luminal B (HER2+) type) case, where the CSCO AI recommended HP (trastuzumab/pattuzumab) regimen and did not recommend combination chemotherapy. In contrast, the guideline experts group recommended the TC+H (docetaxel/cyclophosphamide + trastuzumab) regimen that combines targeted therapy with chemotherapy (**Table S11**). Studies have shown that patients with small tumors of HER2+, LN- still have a higher risk of recurrence compared to patients with small tumors of HER2- (52); for these patients, further chemotherapy can be added to trastuzumab; studies showed that early breast cancer patients with TC+H regimen had 2-year disease-free survival(DFS)and overall survival (OS) rates of 97.8% and 99.2% (52); Therefore, the TC+H regimen can be considered for low-risk patients with T1N0, HER-2-positive disease. This opinion was jointly endorsed by two experts in this trial guideline experts group.

This study has several important strengths. Most importantly, this study developed the first multi-level, multi-indicator system that can comprehensively assess the standardization treatment level of physicians and CDSS, and also provided a methodological basis for the construction of a sensible clinical application model of CDSS and comprehensively assessing the clinical impact of CDSS when it’s implemented in the clinical practice. Second, we have further developed the appropriate connotation of existing indicators that include “Decision Concordance” and “Guideline Conformity”. Thirdly, based on this indicator system, the standardization treatment level and internal variations among different geographical regions were clarified for the first time, and the standardization treatment level of CDSS and its clinical application potential was also examined.

This study still contains several noteworthy limitations. First, the decision-making physicians were all sourced from the same provincial medical region, and further assessment of treatment standardization and regional variation among different provincial medical regions is necessary in the future. Second, this study did not assess the impact of CDSS on physician decision-making.

## 5 Conclusions

This study constructs the first multi-level, multi-indicator system that can more comprehensively assess the standardization level of physicians and CDSS, and provides a methodological basis for constructing a rational clinical practice model of CDSS and assessing the clinical impact of CDSS in a three-dimensional manner. Based on these indicators, we identified that there are significant internal variation in the standardization treatment level of different seniority physicians in different grade hospitals (different geographical regions) in the adjuvant treatment of early breast cancer, and compared with the provincial senior physicians, the standardization treatment level of middle and junior physicians as well as municipal and county hospitals needs to be further improved. CDSS represented by CSCO AI has a higher standardization treatment level than all physicians, and combined with a rational application model, it is expected to provide physicians with immediate decision support and have a positive impact on standardizing physicians’ treatment behaviors. The specific impact of CDSS in improving clinicians’ standardized treatment, balancing medical disparities between different regions, promoting physicians’ own decision-making skills and benefiting patients is yet to be explored in the future, which will establish the overall value of AI-based clinical decision support systems for oncology treatment and will facilitate the formation of best clinical practice models.

## Data availability statement

The original contributions presented in the study are included in the article/**Supplementary Material**. Further inquiries can be directed to the corresponding authors.

## Ethics statement

The study was conducted according to the guidelines of the Declaration of Helsinki, and approved by the Institutional Ethics Committee of the First Affiliated Hospital of Anhui Medical University (protocol code PJ2021-05-07, April 22, 2021). The patients/participants provided their written informed consent to participate in this study.

## Author contributions

Conceptualization, CH. Data curation, CH, JL, YP, CL, XY, XP, XC，MJ, JZ, CL, ZL, DG and JP. Formal analysis, CH, JL, YP, CL，XY，XP and XC. Funding acquisition, ZJ and JP. Investigation, CH, CL, XY, KW, ZS, HL, GJ, FF, TT, SP, LM, XW, YR, ML and FL. Methodology, CH, JL, YP, XY. Project administration, CH, ZJ and JP. Resources, JP. Software, CH, JL, YP. Supervision, ZJ and JP. Validation, CH. Visualization, CH. Writing-original draft, CH. Writing-review & editing, CH, ZJ and JP.
