# Supplementary material for: Assessing the decision quality of artificial intelligence and oncologists of different experience in different regions in breast cancer treatment
Source: Front Oncol. 2023 Jun 9;13:1152013. doi: 10.3389/fonc.2023.1152013 (PMC10289408; doi:10.3389/fonc.2023.1152013)
Supplement: Supplementary file 1 [file DataSheet_1.doc]

Supplementary Material

**Assessing the Decision Quality of Artificial Intelligence and Oncologists of Different Experience in Different Regions in Breast Cancer Treatment**

Chunguang Han ^1^, Yubo Pan ^2^, Chang Liu ^2^, Xiaowei Yang ^2^, Jianbin Li ^3^, Zefei Jiang ^3*^ and Jing Pei ^2*^

*** Correspondence:** Jing Pei, [peijing@ahmu.edu.cn](mailto:peijing@ahmu.edu.cn); Zefei Jiang, [jiangzefei@csco.org.cn](mailto:jiangzefei@csco.org.cn)

**Table S1.** Clinical Characteristics of Each Physician Case Group in the Discordant Subgroup before and after Propensity Score-Matched Analysis

**Table S2.** Decision Concordance between Physicians and CSCO AI

**Table S3.** The Logistic Regression Analysis of Decision Concordance

**Table S4.** Discordance Cases and Reasons of Physician and CDSS Decision-Making

**Table S5.** Concordance with High-Level Physicians

**Table S6.** The Logistic Regression Analysis of Concordance with High-Level Physicians

**Table S7.** Consensus Rate

**Table S8.** Decision Stability

**Table S9.** Guideline Conformity

**Table S10.** The Logistic Regression Analysis of Guideline Conformity

**Table S11.** The Specific Cases and Reasons for Nonconformity with the Guidelines in Different Treatment Stages.

**Supplementary References**

**Table S1. Clinical Characteristics of Each Physician Case Group in the Discordant Subgroup before and after Propensity Score-Matched Analysis**

|  | **Before propensity score matched** | | | | | | | | | | | |
| --- | --- | --- | --- | --- | --- | --- | --- | --- | --- | --- | --- | --- |
|  | **Total**  **(N=1265)** | **Group 101**  **(N=143)** | **Group 102**  **(N=137)** | **Group 103**  **(N=97)** | **Group 201**  **(N=154)** | **Group 202**  **(N=134)** | **Group 203**  **(N=137)** | **Group 301**  **(N=116)** | **Group 302**  **(N=169)** | **Group 303**  **(N=178)** | ***χ*^2^** | ***P*** |
| **Female, n (%)** | 1265 (100.0) | 143 (100.0) | 137 (100.0) | 97 (100.0) | 154 (100.0) | 134 (100.0) | 137 (100.0) | 116 (100.0) | 169 (100.0) | 178 (100.0) |  |  |
| **Age, n (%)** |  |  |  |  |  |  |  |  |  |  | 23.450 | 0.102 |
| ≤44 | 339 (26.8) | 39 (27.3) | 29 (21.2) | 33 (34.0) | 38 (24.7) | 46 (34.3) | 39 (28.5) | 41 (35.3) | 41 (24.3) | 33 (18.5) |  |  |
| 45–54 | 639 (50.5) | 68 (47.6) | 76 (55.5) | 46 (47.4) | 81 (52.6) | 66 (49.3) | 66 (48.2) | 52 (44.8) | 84 (49.7) | 100 (56.2) |  |  |
| ≥55 | 287 (22.7) | 36 (25.2) | 32 (23.4) | 18 (18.6) | 35 (22.7) | 22 (16.4) | 32 (23.4) | 23 (19.8) | 44 (26.0) | 45 (25.3) |  |  |
| **Menstrual status, n (%)** |  |  |  |  |  |  |  |  |  |  | 5.569 | 0.695 |
| Premenopausal | 785 (62.1) | 91 (63.6) | 79 (57.7) | 61 (62.9) | 95 (61.7) | 92 (68.7) | 86 (62.8) | 75 (64.7) | 103 (60.9) | 103 (57.9) |  |  |
| Postmenopausal | 480 (37.9) | 52 (36.4) | 58 (42.3) | 36 (37.1) | 59 (38.3) | 42 (31.3) | 51 (37.2) | 41 (35.3) | 66 (39.1) | 75 (42.1) |  |  |
| **Breast Surgery Modality, n (%)** |  |  |  |  |  |  |  |  |  |  | 12.230 | 0.141 |
| BCS | 306 (24.2) | 28 (19.6) | 38 (27.7) | 26 (26.8) | 35 (22.7) | 35 (26.1) | 26 (19.0) | 39 (33.6) | 42 (24.9) | 37 (20.8) |  |  |
| SM | 959 (75.8) | 115 (80.4) | 99 (72.3) | 71 (73.2) | 119 (77.3) | 99 (73.9) | 111 (81.0) | 77 (66.4) | 127 (75.1) | 141 (79.2) |  |  |
| **Axillary surgery modality, n (%)** |  |  |  |  |  |  |  |  |  |  | 14.979 | 0.060 |
| ALND | 846 (66.9) | 99 (69.2) | 78 (56.9) | 69 (71.1) | 98 (63.6) | 98 (73.1) | 97 (70.8) | 79 (68.1) | 103 (60.9) | 125 (70.2) |  |  |
| SLNB | 419 (33.1) | 44 (30.8) | 59 (43.1) | 28 (28.9) | 56 (36.4) | 36 (26.9) | 40 (29.2) | 37 (31.9) | 66 (39.1) | 53 (29.8) |  |  |
| **TNM Stage, n (%)** |  |  |  |  |  |  |  |  |  |  | 27.467 | 0.037 |
| I | 502 (39.7) | 56 (39.2) | 65 (47.4) | 35 (36.1) | 64 (41.6) | 54 (40.3) | 61 (44.5) | 46 (39.7) | 55 (32.5) | 66 (37.1) |  |  |
| II | 617 (48.8) | 75 (52.4) | 54 (39.4) | 48 (49.5) | 69 (44.8) | 63 (47.0) | 59 (43.1) | 64 (55.2) | 101 (59.8) | 84 (47.2) |  |  |
| III | 146 (11.5) | 12 (8.4) | 18 (13.1) | 14 (14.4) | 21 (13.6) | 17 (12.7) | 17 (12.4) | 6 (5.2) | 13 (7.7) | 28 (15.7) |  |  |
| **Molecular subtype, n (%)** |  |  |  |  |  |  |  |  |  |  | 34.606 | 0.344 |
| HER2 positive HR negative | 129 (10.2) | 16 (11.2) | 11 (8.0) | 13 (13.4) | 9 (5.8) | 17 (12.7) | 9 (6.6) | 16 (13.8) | 23 (13.6) | 15 (8.4) |  |  |
| HER2 positive HR positive | 335 (26.5) | 36 (25.2) | 44 (32.1) | 18 (18.6) | 46 (29.9) | 42 (31.3) | 28 (20.4) | 34 (29.3) | 39 (23.1) | 48 (27.0) |  |  |
| Luminal A | 163 (12.9) | 22 (15.4) | 18 (13.1) | 15 (15.5) | 21 (13.6) | 17 (12.7) | 19 (13.9) | 11 (9.5) | 23 (13.6) | 17 (9.6) |  |  |
| Luminal B (HER2 negative) | 536 (42.4) | 58 (40.6) | 51 (37.2) | 39 (40.2) | 64 (41.6) | 52 (38.8) | 70 (51.1) | 48 (41.4) | 70 (41.4) | 84 (47.2) |  |  |
| TNBC | 102 (8.1) | 11 (7.7) | 13 (9.5) | 12 (12.4) | 14 (9.1) | 6 (4.5) | 11 (8.0) | 7 (6.0) | 14 (8.3) | 14 (7.9) |  |  |
| **Treatment Stage, n (%)** |  |  |  |  |  |  |  |  |  |  | 31.262 | 0.146 |
| **Adjuvant targeted therapy** | 160 (12.6) | 16 (11.2) | 18 (13.1) | 10 (10.3) | 20 (13.0) | 23 (17.2) | 13 (9.5) | 18 (15.5) | 20 (11.8) | 22 (12.4) |  |  |
| Adjuvant chemotherapy | 510 (40.3) | 59 (41.3) | 48 (35.0) | 48 (49.5) | 64 (41.6) | 59 (44.0) | 54 (39.4) | 42 (36.2) | 66 (39.1) | 70 (39.3) |  |  |
| Adjuvant radiotherapy | 315 (24.9) | 39 (27.3) | 44 (32.1) | 19 (19.6) | 39 (25.3) | 24 (17.9) | 35 (25.5) | 20 (17.2) | 39 (23.1) | 56 (31.5) |  |  |
| Adjuvant endocrine therapy | 280 (22.1) | 29 (20.3) | 27 (19.7) | 20 (20.6) | 31 (20.1) | 28 (20.9) | 35 (25.5) | 36 (31.0) | 44 (26.0) | 30 (16.9) |  |  |

**Continued Table**

|  | **After propensity score matched** | | | | | | | | | | | |
| --- | --- | --- | --- | --- | --- | --- | --- | --- | --- | --- | --- | --- |
|  | **Total**  **(N=495)** | **Group 101**  **(N=55)** | **Group 102**  **(N=55)** | **Group 103**  **(N=55)** | **Group 201**  **(N=55)** | **Group 202**  **(N=55)** | **Group 203**  **(N=55)** | **Group 301**  **(N=55)** | **Group 302**  **(N=55)** | **Group 303**  **(N=55)** | ***χ*^2^** | ***P*** |
| **Female, n (%)** | 495 (100.0) | 55 (100.0) | 55 (100.0) | 55 (100.0) | 55 (100.0) | 55 (100.0) | 55 (100.0) | 55 (100.0) | 55 (100.0) | 55 (100.0) |  |  |
| **Age, n (%)** |  |  |  |  |  |  |  |  |  |  | 16.600 | 0.412 |
| ≤44 | 141 (28.5) | 19 (34.5) | 19 (34.5) | 14 (25.5) | 13 (23.6) | 16 (29.1) | 16 (29.1) | 16 (29.1) | 17 (30.9) | 11 (20.0) |  |  |
| 45–54 | 266 (53.7) | 24 (43.6) | 32 (58.2) | 31 (56.4) | 28 (50.9) | 28 (50.9) | 34 (61.8) | 26 (47.3) | 28 (50.9) | 35 (63.6) |  |  |
| ≥55 | 88 (17.8) | 12 (21.8) | 4 (7.3) | 10 (18.2) | 14 (25.5) | 11 (20.0) | 5 (9.1) | 13 (23.6) | 10 (18.2) | 9 (16.4) |  |  |
| **Menstrual status, n (%)** |  |  |  |  |  |  |  |  |  |  | 8.018 | 0.432 |
| Premenopausal | 317 (64.0) | 36 (65.5) | 41 (74.5) | 34 (61.8) | 31 (56.4) | 37 (67.3) | 40 (72.7) | 32 (58.2) | 32 (58.2) | 34 (61.8) |  |  |
| Postmenopausal | 178 (36.0) | 19 (34.5) | 14 (25.5) | 21 (38.2) | 24 (43.6) | 18 (32.7) | 15 (27.3) | 23 (41.8) | 23 (41.8) | 21 (38.2) |  |  |
| **Breast Surgery Modality, n (%)** |  |  |  |  |  |  |  |  |  |  | 2.970 | 0.936 |
| BCS | 120 (24.2) | 13 (23.6) | 13 (23.6) | 13 (23.6) | 16 (29.1) | 12 (21.8) | 11 (20.0) | 11 (20.0) | 15 (27.3) | 16 (29.1) |  |  |
| SM | 375 (75.8) | 42 (76.4) | 42 (76.4) | 42 (76.4) | 39 (70.9) | 43 (78.2) | 44 (80.0) | 44 (80.0) | 40 (72.7) | 39 (70.9) |  |  |
| **Axillary surgery modality, n (%)** |  |  |  |  |  |  |  |  |  |  | 2.312 | 0.970 |
| ALND | 333 (67.3) | 36 (65.5) | 35 (63.6) | 38 (69.1) | 38 (69.1) | 38 (69.1) | 40 (72.7) | 38 (69.1) | 36 (65.5) | 34 (61.8) |  |  |
| SLNB | 162 (32.7) | 19 (34.5) | 20 (36.4) | 17 (30.9) | 17 (30.9) | 17 (30.9) | 15 (27.3) | 17 (30.9) | 19 (34.5) | 21 (38.2) |  |  |
| **TNM Stage, n (%)** |  |  |  |  |  |  |  |  |  |  | 22.565 | 0.126 |
| I | 193 (39.0) | 17 (30.9) | 22 (40.0) | 21 (38.2) | 20 (36.4) | 22 (40.0) | 23 (41.8) | 22 (40.0) | 18 (32.7) | 28 (50.9) |  |  |
| II | 247 (49.9) | 34 (61.8) | 26 (47.3) | 29 (52.7) | 28 (50.9) | 22 (40.0) | 23 (41.8) | 30 (54.5) | 35 (63.6) | 20 (36.4) |  |  |
| III | 55 (11.1) | 4 (7.3) | 7 (12.7) | 5 (9.1) | 7 (12.7) | 11 (20.0) | 9 (16.4) | 3 (5.5) | 2 (3.6) | 7 (12.7) |  |  |
| **Molecular subtype, n (%)** |  |  |  |  |  |  |  |  |  |  | 20.852 | 0.935 |
| HER2 positive HR negative | 46 (9.3) | 7 (12.7) | 4 (7.3) | 6 (10.9) | 2 (3.6) | 4 (7.3) | 5 (9.1) | 8 (14.5) | 6 (10.9) | 4 (7.3) |  |  |
| HER2 positive HR positive | 126 (25.5) | 10 (18.2) | 19 (34.5) | 11 (20.0) | 19 (34.5) | 13 (23.6) | 12 (21.8) | 15 (27.3) | 15 (27.3) | 12 (21.8) |  |  |
| Luminal A | 66 (13.3) | 8 (14.5) | 7 (12.7) | 10 (18.2) | 6 (10.9) | 7 (12.7) | 7 (12.7) | 6 (10.9) | 10 (18.2) | 5 (9.1) |  |  |
| Luminal B (HER2 negative) | 220 (44.4) | 26 (47.3) | 21 (38.2) | 23 (41.8) | 26 (47.3) | 27 (49.1) | 28 (50.9) | 22 (40.0) | 19 (34.5) | 28 (50.9) |  |  |
| TNBC | 37 (7.5) | 4 (7.3) | 4 (7.3) | 5 (9.1) | 2 (3.6) | 4 (7.3) | 3 (5.5) | 4 (7.3) | 5 (9.1) | 6 (10.9) |  |  |
| **Treatment Stage, n (%)** |  |  |  |  |  |  |  |  |  |  | 24.966 | 0.408 |
| **Adjuvant targeted therapy** | 64 (12.9) | 4 (7.3) | 8 (14.5) | 5 (9.1) | 8 (14.5) | 7 (12.7) | 7 (12.7) | 12 (21.8) | 6 (10.9) | 7 (12.7) |  |  |
| Adjuvant chemotherapy | 214 (43.2) | 25 (45.5) | 17 (30.9) | 29 (52.7) | 24 (43.6) | 23 (41.8) | 21 (38.2) | 26 (47.3) | 25 (45.5) | 24 (43.6) |  |  |
| Adjuvant radiotherapy | 109 (22.0) | 18 (32.7) | 18 (32.7) | 8 (14.5) | 12 (21.8) | 11 (20.0) | 15 (27.3) | 4 (7.3) | 11 (20.0) | 12 (21.8) |  |  |
| Adjuvant endocrine therapy | 108 (21.8) | 8 (14.5) | 12 (21.8) | 13 (23.6) | 11 (20.0) | 14 (25.5) | 12 (21.8) | 13 (23.6) | 13 (23.6) | 12 (21.8) |  |  |

*P* values are from Chi-square test of differences across different Case Groups. HER2=human epidermal growth factor receptor 2. HR=hormone receptor. Luminal A=HER2 negative HR positive, and PR≥20% and Ki-67<15%. Luminal B (HER2 negative) =HER2 negative HR positive, and PR < 20% or Ki-67≥15%. TNBC=triple-negative breast cancer (HR and HER2 negative-tumors). PR=progesterone receptor. Ki-67=proliferating cell nuclear antigen-67. BCS=[breast conserving surgery](https://pubmed.ncbi.nlm.nih.gov/31176055/). SM=simple mastectomy. SLNB=sentinel lymph node biopsy. ALND=axillary lymph node dissection.

**Table S2. Decision Concordance between Physicians and CSCO AI**

|  | **All physician' decision concordance (before calibration)** | | | **All physician' decision concordance**  **(after calibration)** | | |
| --- | --- | --- | --- | --- | --- | --- |
|  | **Concordant cases, n (%)** | **Non-concordant cases, n (%)** | ***P*** | **Concordant cases, n (%)** | **Non-concordant cases, n (%)** | ***P*** |
| **Overall** (N=6372) | 5107 (80.1) | 1265 (19.9) |  | 5107 (80.1) | 1265 (19.9) |  |
| **Different Seniority Physicians** |  |  | 0.474 |  |  | **<0.001** |
| **Senior Physician (N=2124)** | 1711 (80.6) | 413 (19.4) |  | 1711 (80.6) | 413 (19.4) |  |
| Middle Physician (N=2124) | 1684 (79.3) | 440 (20.7) |  | 1781 (83.8) | 343 (16.2) |  |
| Junior Physician (N=2124) | 1712 (80.6) | 412 (19.4) |  | 1466 (69.0) | 658 (31.0) |  |
| **Different Grade Hospitals** |  |  | **0.004** |  |  | **<0.001** |
| Provincial Hospital (N=2124) | 1661 (78.2) | 463 (21.8) |  | 1661 (78.2) | 463 (21.8) |  |
| Municipal Hospital (N=2124) | 1699 (80.0) | 425 (20.0) |  | 1105 (52.0) | 1019 (48.0) |  |
| County Hospital (N=2124) | 1747 (82.3) | 377 (17.7) |  | 1200 (56.5) | 924 (43.5) |  |
| **Different Seniority Physicians in Different Grades Hospitals** |  |  | **<0.001** |  |  | **<0.001** |
| 301 Provincial Senior (N=708) | 592 (83.6) | 116 (16.4) |  | 572 (80.9) | 136 (19.1) |  |
| 302 Provincial Middle (N=708) | 539 (76.1) | 169 (23.9) |  | 539 (76.1) | 169 (23.9) |  |
| 303 Provincial Junior (N=708) | 530 (74.9) | 178 (25.1) |  | 477 (67.4) | 231 (32.6) |  |
| 201 Municipal Senior (N=708) | 554 (78.2) | 154 (21.8) |  | 388 (54.8) | 320 (45.2) |  |
| 202 Municipal Middle (N=708) | 574 (81.1) | 134 (18.9) |  | 269 (37.9) | 439 (62.1) |  |
| 203 Municipal Junior (N=708) | 571 (80.6) | 137 (19.4) |  | 400 (56.5) | 308 (43.5) |  |
| 101 County Senior (N=708) | 565 (79.8) | 143 (20.2) |  | 358 (50.5) | 350 (49.5) |  |
| 102 County Middle (N=708) | 571 (80.6) | 137 (19.4) |  | 552 (78.0) | 156 (22.0) |  |
| 103 County Junior (N=708) | 611 (86.3) | 97 (13.7) |  | 224 (31.7) | 484 (68.3) |  |
| **Physicians** |  |  | **<0.001** |  |  | NA |
| 3011 Provincial Senior1 (N=236) | 180 (76.3) | 56 (23.7) |  | NA | NA |  |
| 3012 Provincial Senior2 (N=236) | 216 (91.5) | 20 (8.5) |  | NA | NA |  |
| 3013 Provincial Senior3 (N=236) | 196 (83.1) | 40 (16.9) |  | NA | NA |  |
| 3021 Provincial Middle1 (N=236) | 196 (83.1) | 40 (16.9) |  | NA | NA |  |
| 3022 Provincial Middle2 (N=236) | 162 (68.6) | 74 (31.4) |  | NA | NA |  |
| 3023 Provincial Middle3 (N=236) | 181 (76.7) | 55 (23.3) |  | NA | NA |  |
| 3031 Provincial Junior1 (N=236) | 188 (79.7) | 48 (20.3) |  | NA | NA |  |
| 3032 Provincial Junior2 (N=236) | 181 (76.7) | 55 (23.3) |  | NA | NA |  |
| 3034 Provincial Junior4 (N=236) | 161 (68.2) | 75 (31.8) |  | NA | NA |  |
| 2011 Municipal Senior1 (N=236) | 202 (85.6) | 34 (14.4) |  | NA | NA |  |
| 2012 Municipal Senior2 (N=236) | 192 (81.4) | 44 (18.6) |  | NA | NA |  |
| 2013 Municipal Senior3 (N=236) | 160 (67.8) | 76 (32.2) |  | NA | NA |  |
| 2021 Municipal Middle1 (N=236) | 191 (80.9) | 45 (19.1) |  | NA | NA |  |
| 2022 Municipal Middle2 (N=236) | 180 (76.3) | 56 (23.7) |  | NA | NA |  |
| 2023 Municipal Middle3 (N=236) | 203 (86.0) | 33 (14.0) |  | NA | NA |  |
| 2031 Municipal Junior1 (N=236) | 211 (89.4) | 25 (10.6) |  | NA | NA |  |
| 2032 Municipal Junior2 (N=236) | 148 (62.7) | 88 (37.3) |  | NA | NA |  |
| 2033 Municipal Junior3 (N=236) | 212 (89.8) | 24 (10.2) |  | NA | NA |  |
| 1011 County Senior1 (N=236) | 174 (73.7) | 62 (26.3) |  | NA | NA |  |
| 1012 County Senior2 (N=236) | 182 (77.1) | 54 (22.9) |  | NA | NA |  |
| 1013 County Senior3 (N=236) | 209 (88.6) | 27 (11.4) |  | NA | NA |  |
| 1021 County Middle1 (N=236) | 212 (89.8) | 24 (10.2) |  | NA | NA |  |
| 1022 County Middle2 (N=236) | 176 (74.6) | 60 (25.4) |  | NA | NA |  |
| 1023 County Middle3 (N=236) | 183 (77.5) | 53 (22.5) |  | NA | NA |  |
| 1031 County Junior1 (N=236) | 198 (83.9) | 38 (16.1) |  | NA | NA |  |
| 1032 County Junior2 (N=236) | 220 (93.2) | 16 (6.8) |  | NA | NA |  |
| 1033 County Junior3 (N=236) | 193 (81.8) | 43 (18.2) |  | NA | NA |  |
| **Age** |  |  | 0.062 |  |  | **<0.001** |
| ≤44 (N=1716) | 1377 (80.2) | 339 (19.8) |  | 1210 (70.5) | 506 (29.5) |  |
| 45–54 (N=3060) | 2421 (79.1) | 639 (20.9) |  | 2421 (79.1) | 639 (20.9) |  |
| ≥55 (N=1596) | 1309 (82.0) | 287 (18.0) |  | 938 (58.7) | 658 (41.3) |  |
| **Menstrual status** |  |  | 0.108 |  |  | **<0.001** |
| Premenopausal (N=3828) | 3043 (79.5) | 785 (20.5) |  | 3043 (79.5) | 785 (20.5) |  |
| Postmenopausal (N=2544) | 2064 (81.1) | 480 (18.9) |  | 1455 (57.2) | 1089 (42.8) |  |
| **Breast Surgery Modality** |  |  | **<0.001** |  |  | **<0.001** |
| BCS (N=1152) | 846 (73.4) | 306 (26.6) |  | 807 (70.0) | 345 (30.0) |  |
| SM (N=5220) | 4261 (81.6) | 959 (18.4) |  | 4261 (81.6) | 959 (18.4) |  |
| **Axillary surgery modality** |  |  | **<0.001** |  |  | **<0.001** |
| **ALND (N=4548)** | 3702 (81.4) | 846 (18.6) |  | 3702 (81.4) | 846 (18.6) |  |
| SLNB (N=1824) | 1405 (77.0) | 419 (23.0) |  | 1381 (75.7) | 443 (24.3) |  |
| **TNM Stage** |  |  | **<0.001** |  |  | **<0.001** |
| I (N=2292) | 1790 (78.1) | 502 (21.9) |  | 1568 (68.4) | 724 (31.6) |  |
| II (N=3180) | 2563 (80.6) | 617 (19.4) |  | 1921 (60.4) | 1259 (39.6) |  |
| **III (N=900)** | 754 (83.8) | 146 (16.2) |  | 754 (83.8) | 146 (16.2) |  |
| **Molecular subtype** |  |  | **<0.001** |  |  | **<0.001** |
| HER2 positive HR negative (N=576) | 447 (77.6) | 129 (22.4) |  | 363 (63.1) | 213 (36.9) |  |
| HER2 positive HR positive (N=1332) | 997 (74.8) | 335 (25.2) |  | 662 (49.7) | 670 (50.3) |  |
| Luminal A (N=972) | 809 (83.2) | 163 (16.8) |  | 836 (86.0) | 136 (14.0) |  |
| Luminal B (HER2 negative) (N=2676) | 2140 (80.0) | 536 (20.0) |  | 2140 (80.0) | 536 (20.0) |  |
| **TNBC (N=816)** | 714 (87.5) | 102 (12.5) |  | 636 (77.9) | 180 (22.1) |  |
| **Treatment Stage** |  |  | **<0.001** |  |  | **<0.001** |
| **Adjuvant targeted therapy (N=1593)** | 1433 (90.0) | 160 (10.0) |  | 792 (49.7) | 801 (50.3) |  |
| Adjuvant chemotherapy (N=1593) | 1083 (68.0) | 510 (32.0) |  | 853 (53.6) | 740 (46.4) |  |
| Adjuvant radiotherapy (N=1593) | 1278 (80.2) | 315 (19.8) |  | 1243 (78.1) | 350 (21.9) |  |
| Adjuvant endocrine therapy (N=1593) | 1313 (82.4) | 280 (17.6) |  | 1313 (82.4) | 280 (17.6) |  |

NA=not applicable. HER2=human epidermal growth factor receptor 2. HR=hormone receptor. Luminal A=HER2 negative HR positive, and PR≥20% and Ki-67<15%. Luminal B (HER2 negative) =HER2 negative HR positive, and PR<20% or Ki-67≥15%. TNBC=triple-negative breast cancer (HR and HER2 negative-tumors). PR=progesterone receptor. Ki-67=proliferating cell nuclear antigen-67. BCS=[breast conserving surgery](https://pubmed.ncbi.nlm.nih.gov/31176055/). SM=simple mastectomy. SLNB=sentinel lymph node biopsy. ALND=axillary lymph node dissection.

**Table S3. The Logistic Regression Analysis of Decision Concordance**

|  | **All physician' decision concordance**  **-Model 1** | | **All physician' decision concordance**  **-Model 2** | |
| --- | --- | --- | --- | --- |
|  | **OR（95% Cl）** | ***P*** | **OR（95% Cl）** | ***P*** |
| **Overall** (N=6372) |  |  |  |  |
| **Different Seniority Physicians** |  | 0.446 |  | NA |
| **Senior Physician (N=2124)** | 1.000 (1.000-1.000) | Reference | NA | NA |
| Middle Physician (N=2124) | 0.918 (0.786-1.072) | 0.280 | NA | NA |
| Junior Physician (N=2124) | 1.002 (0.857-1.173) | 0.976 | NA | NA |
| **Different Grade Hospitals** |  | **0.003** |  | NA |
| Provincial Hospital (N=2124) | 1.000 (1.000-1.000) | Reference | NA | NA |
| Municipal Hospital (N=2124) | 1.123 (0.964-1.308) | 0.138 | NA | NA |
| County Hospital (N=2124) | 1.314 (1.123-1.536) | **0.001** | NA | NA |
| **Different Seniority Physicians in Different Grades of Hospitals** |  | NA |  | **<0.001** |
| 301 Provincial Senior (N=708) | NA | NA | 1.000 (1.000-1.000) | Reference |
| 302 Provincial Middle (N=708) | NA | NA | 0.586 (0.446-0.770) | **<0.001** |
| 303 Provincial Junior (N=708) | NA | NA | 0.558 (0.425-0.732) | **<0.001** |
| 201 Municipal Senior (N=708) | NA | NA | 0.684 (0.518-0.902) | **0.007** |
| 202 Municipal Middle (N=708) | NA | NA | 0.831 (0.627-1.101) | 0.197 |
| 203 Municipal Junior (N=708) | NA | NA | 0.779 (0.588-1.033) | 0.083 |
| 101 County Senior (N=708) | NA | NA | 0.736 (0.557-0.974) | **0.032** |
| 102 County Middle (N=708) | NA | NA | 0.800 (0.604-1.061) | 0.122 |
| 103 County Junior (N=708) | NA | NA | 1.248 (0.925-1.685) | 0.148 |
| **Age** |  | 0.168 |  | 0.226 |
| ≤44 (N=1716) | 1.000 (1.000-1.000) | Reference | 1.000 (1.000-1.000) | Reference |
| 45–54 (N=3060) | 0.868 (0.733-1.027) | 0.099 | 0.893 (0.753-1.058) | 0.192 |
| ≥55 (N=1596) | 0.955 (0.737-1.237) | 0.727 | 1.003 (0.773-1.302) | 0.981 |
| **Menstrual status** |  | 0.262 |  | 0.377 |
| Premenopausal (N=3828) | 1.000 (1.000-1.000) | Reference | 1.000 (1.000-1.000) | Reference |
| Postmenopausal (N=2544) | 1.113 (0.923-1.342) | 0.262 | 1.088 (0.902-1.312) | 0.377 |
| **Breast Surgery Modality** |  | **<0.001** |  | **<0.001** |
| BCS (N=1152) | 1.000 (1.000-1.000) | Reference | 1.000 (1.000-1.000) | Reference |
| SM (N=5220) | 1.651 (1.387-1.966) | **<0.001** | 1.723 (1.444-2.055) | **<0.001** |
| **Axillary surgery modality** |  | 0.926 |  | 0.726 |
| **ALND (N=4548)** | 1.000 (1.000-1.000) | Reference | 1.000 (1.000-1.000) | Reference |
| SLNB (N=1824) | 0.992 (0.842-1.169) | 0.926 | 1.030 (0.873-1.215) | 0.726 |
| **TNM Stage** |  | **0.002** |  | **0.004** |
| I (N=2292) | 0.685 (0.547-0.857) | **0.001** | 0.691 (0.551-0.865) | **0.001** |
| II (N=3180) | 0.815 (0.663-1.003) | 0.053 | 0.808 (0.657-0.995) | **0.045** |
| **III (N=900)** | 1.000 (1.000-1.000) | Reference | 1.000 (1.000-1.000) | Reference |
| **Molecular subtype** |  | **<0.001** |  | **<0.001** |
| HER2 positive HR negative (N=576) | 0.455 (0.339-0.611) | **<0.001** | 0.435 (0.323-0.585) | **<0.001** |
| HER2 positive HR positive (N=1332) | 0.391 (0.305-0.502) | **<0.001** | 0.384 (0.299-0.492) | **<0.001** |
| Luminal A (N=972) | 0.728 (0.553-0.960) | **0.024** | 0.698 (0.529-0.921) | **0.011** |
| Luminal B (HER2 negative) (N=2676) | 0.557 (0.441-0.704) | **<0.001** | 0.547 (0.432-0.691) | **<0.001** |
| **TNBC (N=816)** | 1.000 (1.000-1.000) | Reference | 1.000 (1.000-1.000) | Reference |
| **Treatment Stage** |  | **<0.001** |  | **<0.001** |
| **Adjuvant targeted therapy (N=1593)** | 1.000 (1.000-1.000) | Reference | 1.000 (1.000-1.000) | Reference |
| Adjuvant chemotherapy (N=1593) | 0.229 (0.188-0.279) | **<0.001** | 0.227 (0.187-0.277) | **<0.001** |
| Adjuvant radiotherapy (N=1593) | 0.446 (0.363-0.549) | **<0.001** | 0.445 (0.361-0.547) | **<0.001** |
| Adjuvant endocrine therapy (N=1593) | 0.518 (0.420-0.639) | **<0.001** | 0.516 (0.418-0.637) | **<0.001** |
| **Constant** | 13.173 | **<0.001** | 18.296 | **<0.001** |

NA=not applicable. HER2=human epidermal growth factor receptor 2. HR=hormone receptor. Luminal A=HER2 negative HR positive, and PR≥20% and Ki-67<15%. Luminal B (HER2 negative) =HER2 negative HR positive, and PR<20% or Ki-67≥15%. TNBC=triple-negative breast cancer (HR and HER2 negative-tumors). PR=progesterone receptor. Ki-67=proliferating cell nuclear antigen-67. BCS=[breast conserving surgery](https://pubmed.ncbi.nlm.nih.gov/31176055/). SM=simple mastectomy. SLNB=sentinel lymph node biopsy. ALND=axillary lymph node dissection.

**Table S4. Discordance Cases and Reasons of Physician and CDSS Decision-Making**

| **Cases and Treatment Decisions** | **Reasons** |
| --- | --- |
| **[Case one:124-3032†]** Patient, female, 50 years old, premenopausal. 2020-10-15 "left breast cancer breast conserving surgery + SLNB" Showed: cancer size 1·2*1·2*1cm, invasive ductal carcinoma, WHO grade II, with intra-ductal carcinoma; ER (1-2+, 80%), PR ( 1-2+, 80%), HER-2 (2+/FISH-), KI-67 (20%); SLN (0/2).  **[Treatment Decision]** CDSS Not recommended vs. Physician AC | **Treatment strategy differences:** The physician prefers the AC program or could consider further polygenic testing to support the evaluation; and retains the original opinion after referring to the CDSS's opinion. |
| **[Case two:216-3022†]** Patient, female, 42 years old, premenopausal. 2020-06-02 " Right breast cancer breast-conserving surgery+SLNB", showed: cancer size 2·2*2*1·6 cm, invasive carcinoma, non-specific type, WHO grade II-III; ER (-), PR (-), HER-2 (2+/FISH-), KI-67 (60%). SLN (0/1).  **[Treatment Decision]** CDSS AC-T vs. Physician TC | **Treatment strategy differences:** physicians made a decision change to accept the CDSS-recommended AC-T program after referring to the CDSS opinion; physicians considered the evidence was more robust for the program provided by the CDSS. |
| **[ Case three:565-3032†]** Patient, female, 71 years old, postmenopausal. 2020-09-10 " Left breast cancer modified radical surgery" showed: cancer size 2·0*1·3*1·2cm; invasive cancer, non-specific type, WHO grade II; no definite vascular and nerve invasion; 16 lymph nodes were detected in the ipsilateral axilla without cancer metastasis; an additional cancer node with a diameter of 0·8cm was seen. ER (90% ), PR (90% ), Her-2 (2+), FISH (-), Ki-67 (10% ).  **[Treatment Decision]** CDSS AC/TC vs. Physician Not recommended | **Missing or misreading information:** the physician had omitted the important medical information of “an additional cancer node” and did not recommend the patient to receive chemotherapy in the initial decision, and after being prompted by CDSS, the physician reviewed the case and realized the problem, and finally adopted AC program consistent with the CDSS level I recommendations. |
| **[Case four:340-3022†]** Patient, female, 58 years old, postmenopausal. 2020-12-01 "Left breast simple mastectomy + SLNB" showed: cancer size 4*3*2·8 cm; invasive carcinoma, non-specific type, WHO grade III, vascular tumor embolus (+); ER(-), PR(-), HER-2(1+), Ki-67 (+20%); SLN (0/4).  **[Treatment Decision]** CDSS AC-T vs. Physician TAC | **Incorrect concordance assessment:** The physician incorrectly assesses the consistency of his own and the CDSS's decision opinions as concordance. |

**†** The number is the relevant case' and the decision-making physician' ID number. AC: anthracycline combined with cyclophosphamide. TC=docetaxel combined with cyclophosphamide. AC-T=anthracycline combined with cyclophosphamide sequential paclitaxel. TAC=docetaxel combined with doxorubicin and cyclophosphamide.

**Table S5. Concordance with High-Level Physicians**

| **Variable** | **G7-**  **1033 /2022/CSCO A I *vs.* 3011** | | | **G8-**  **1031/2021/CSCO A I *vs.* 3013** | | | **G9-**  **1032 /2023/CSCO A I *vs.* 3012** | | |
| --- | --- | --- | --- | --- | --- | --- | --- | --- | --- |
|  | **N** | **Concordant cases, n (%)** | ***P*** | **N** | **Concordant cases, n (%)** | ***P*** | **N** | **Concordant cases, n (%)** | ***P*** |
| **Overall** | 708 | 526 (74.3) |  | 708 | 548 (77.4) |  | 708 | 625 (88.3) |  |
| **Different Seniority of Physicians** |  |  | .488 |  |  | **.036** |  |  | .127 |
| G7-1033/G8-1031/G9-1032 | 236 | 177 (75.0) |  | 236 | 174 (73.7) |  | 236 | 207 (87.7) |  |
| G7-2022/G8-2021/G9-2023 | 236 | 169 (71.6) |  | 236 | 178 (75.4) |  | 236 | 202 (85.6) |  |
| CSCO AI | 236 | 180 (76.3) |  | 236 | 196 (83.1) |  | 236 | 216 (91.5) |  |
| **Age** |  |  | **< .001** |  |  | **.024** |  |  | .760 |
| ≤44 | 204 | 132 (64.7) |  | 252 | 206 (81.7) |  | 204 | 178 (87.3) |  |
| 45–54 | 360 | 276 (76.7) |  | 252 | 181 (71.8) |  | 384 | 339 (88.3) |  |
| ≥55 | 144 | 118 (81.9) |  | 204 | 161 (78.9) |  | 120 | 108 (90.0) |  |
| **Menstrual status** |  |  | **.008** |  |  | .083 |  |  | .078 |
| Premenopausal | 420 | 297 (70.7) |  | 444 | 353 (79.5) |  | 432 | 374 (86.6) |  |
| Postmenopausal | 288 | 229 (79.5) |  | 264 | 195 (73.9) |  | 276 | 251 (90.9) |  |
| **Breast Surgery Modality** |  |  | **< .001** |  |  | **< .001** |  |  | .097 |
| BCS | 156 | 96 (61.5) |  | 156 | 102 (65.4) |  | 132 | 111 (84.1) |  |
| SM | 552 | 430 (77.9) |  | 552 | 446 (80.8) |  | 576 | 514 (89.2) |  |
| **Axillary surgery modality** |  |  | .810 |  |  | **.001** |  |  | .897 |
| **ALND** | 540 | 400 (74.1) |  | 540 | 434 (80.4) |  | 516 | 456 (88.4) |  |
| SLNB | 168 | 126 (75.0) |  | 168 | 114 (67.9) |  | 192 | 169 (88.0) |  |
| **TNM Stage** |  |  | **.049** |  |  | **.014** |  |  | .859 |
| I | 228 | 157 (68.9) |  | 228 | 164 (71.9) |  | 228 | 202 (88.6) |  |
| II | 420 | 320 (76.2) |  | 396 | 311 (78.5) |  | 288 | 252 (87.5) |  |
| **III** | 60 | 49 (81.7) |  | 84 | 73 (86.9) |  | 192 | 171 (89.1) |  |
| **Molecular subtype** |  |  | **< .001** |  |  | **.003** |  |  | **.026** |
| HER2 positive HR negative | 84 | 57 (67.9) |  | 84 | 64 (76.2) |  | 48 | 43 (89.6) |  |
| HER2 positive HR positive | 108 | 54 (50.0) |  | 168 | 113 (67.3) |  | 168 | 146 (86.9) |  |
| Luminal A | 60 | 49 (81.7) |  | 96 | 74 (77.1) |  | 192 | 172 (89.6) |  |
| Luminal B (HER2 negative) | 384 | 304 (79.2) |  | 264 | 215 (81.4) |  | 228 | 193 (84.6) |  |
| **TNBC** | 72 | 62 (86.1) |  | 96 | 82 (85.4) |  | 72 | 71 (98.6) |  |
| **Treatment Stage** |  |  | **< .001** |  |  | **< .001** |  |  | **.003** |
| **Adjuvant targeted therapy** | 177 | 146 (82.5) |  | 177 | 153 (86.4) |  | 177 | 169 (95.5) |  |
| Adjuvant chemotherapy | 177 | 109 (61.6) |  | 177 | 124 (70.1) |  | 177 | 149 (84.2) |  |
| Adjuvant radiotherapy | 177 | 147 (83.1) |  | 177 | 144 (81.4) |  | 177 | 157 (88.7) |  |
| Adjuvant endocrine therapy | 177 | 124 (70.1) |  | 177 | 127 (71.8) |  | 177 | 150 (84.7) |  |

Abbreviations: HER2=human epidermal growth factor receptor 2. HR=hormone receptor. Luminal A=HER2 negative HR positive, and PR≥20% and Ki-67<15%. Luminal B (HER2 negative) =HER2 negative HR positive, and PR<20% or Ki-67≥15%. TNBC=triple-negative breast cancer (HR and HER2 negative-tumors). PR=progesterone receptor. Ki-67=proliferating cell nuclear antigen-67. BCS=[breast conserving surgery](https://pubmed.ncbi.nlm.nih.gov/31176055/). SM=simple mastectomy. SLNB=sentinel lymph node biopsy. ALND=axillary lymph node dissection.

**Table S6. The Logistic Regression Analysis of Concordance with High-Level Physicians**

|  | **G7-**  **1033 /2022/CSCO AI *vs.* 3011** | | **G8-**  **1031/2021/CSCO AI *vs.* 3013** | | **G9-**  **1032 /2023/CSCO AI *vs.* 3012** | |
| --- | --- | --- | --- | --- | --- | --- |
|  | **OR（95% Cl）** | ***P*** | **OR（95% Cl）** | ***P*** | **OR（95% Cl）** | ***P*** |
| **Overall** |  |  |  |  |  |  |
| **Different Decision Makers** |  | 0.428 |  | **0.026** |  | 0.118 |
| G7-1033/G8-1031/G9-1032 | 0.921 (0.583-1.456) | 0.726 | 0.537 (0.335-0.862) | **0.010** | 0.648 (0.350-1.198) | 0.167 |
| G7-2022/G8-2021/G9-2023 | 0.750 (0.478-1.175) | 0.209 | 0.594 (0.369-0.956) | **0.032** | 0.533 (0.293-0.971) | **0.040** |
| CSCO AI | 1.000 (1.000-1.000) | Reference | 1.000 (1.000-1.000) | Reference | 1.000 (1.000-1.000) | Reference |
| **Age** |  | 0.189 |  | **0.006** |  | 0.588 |
| ≤44 | 1.000 (1.000-1.000) | Reference | 1.000 (1.000-1.000) | Reference | 1.000 (1.000-1.000) | Reference |
| 45–54 | 1.558 (0.940-2.582) | 0.085 | 0.753 (0.450-1.262) | 0.282 | 0.750 (0.382-1.471) | 0.403 |
| ≥55 | 1.965 (0.831-4.647) | 0.124 | 2.176 (0.933-5.075) | 0.072 | 0.570 (0.187-1.731) | 0.321 |
| **Menstrual status** |  | 0.268 |  | **0.012** |  | **0.033** |
| Premenopausal | 1.000 (1.000-1.000) | Reference | 1.000 (1.000-1.000) | Reference | 1.000 (1.000-1.000) | Reference |
| Postmenopausal | 1.391 (0.776-2.495) | 0.268 | 0.437 (0.228-0.837) | **0.012** | 2.354 (1.072-5.171) | **0.033** |
| **Breast Surgery Modality** |  | **0.004** |  | **0.001** |  | 0.114 |
| BCS | 1.000 (1.000-1.000) | Reference | 1.000 (1.000-1.000) | Reference | 1.000 (1.000-1.000) | Reference |
| SM | 1.961 (1.242-3.096) | **0.004** | 2.974 (1.575-5.619) | **0.001** | 1.760 (0.872-3.552) | 0.114 |
| **Axillary surgery modality** |  | 0.278 |  | 0.385 |  | 0.827 |
| **ALND** | 1.000 (1.000-1.000) | Reference | 1.000 (1.000-1.000) | Reference | 1.000 (1.000-1.000) | Reference |
| SLNB | 1.314 (0.803-2.150) | 0.278 | 1.355 (0.683-2.687) | 0.385 | 0.927 (0.472-1.823) | 0.827 |
| **TNM Stage** |  | **0.005** |  | **0.019** |  | 0.509 |
| I | 0.589 (0.269-1.291) | 0.186 | 0.297 (0.126-0.698) | **0.005** | 0.676 (0.328-1.393) | 0.288 |
| II | 1.185 (0.554-2.534) | 0.662 | 0.451 (0.212-0.960) | **0.039** | 0.938 (0.483-1.822) | 0.850 |
| **III** | 1.000 (1.000-1.000) | Reference | 1.000 (1.000-1.000) | Reference | 1.000 (1.000-1.000) | Reference |
| **Molecular subtype** |  | **<0.001** |  | **0.010** |  | **0.043** |
| HER2 positive HR negative | 0.292 (0.116-0.732) | **0.009** | 0.388 (0.169-0.891) | **0.026** | 0.064 (0.007-0.611) | **0.017** |
| HER2 positive HR positive | 0.174 (0.074-0.410) | **<0.001** | 0.410 (0.200-0.837) | **0.014** | 0.065 (0.008-0.509) | **0.009** |
| Luminal A | 0.625 (0.232-1.685) | 0.353 | 0.951 (0.416-2.176) | 0.906 | 0.140 (0.018-1.090) | 0.060 |
| Luminal B (HER2 negative) | 0.746 (0.350-1.592) | 0.449 | 0.781 (0.393-1.553) | 0.481 | 0.077 (0.010-0.585) | **0.013** |
| **TNBC** | 1.000 (1.000-1.000) | Reference | 1.000 (1.000-1.000) | Reference | 1.000 (1.000-1.000) | Reference |
| **Treatment Stage** |  | **<0.001** |  | **<0.001** |  | **0.005** |
| **Adjuvant targeted therapy** | 1.000 (1.000-1.000) | Reference | 1.000 (1.000-1.000) | Reference | 1.000 (1.000-1.000) | Reference |
| Adjuvant chemotherapy | 0.292 (0.172-0.495) | **<0.001** | 0.334 (0.190-0.586) | **<0.001** | 0.242 (0.106-0.553) | **0.001** |
| Adjuvant radiotherapy | 1.046 (0.582-1.877) | 0.881 | 0.664 (0.366-1.204) | 0.177 | 0.363 (0.154-0.855) | **0.020** |
| Adjuvant endocrine therapy | 0.451 (0.263-0.772) | **0.004** | 0.366 (0.208-0.644) | **<0.001** | 0.253 (0.111-0.580) | **0.001** |
| **Constant** | 4.358 | **0.013** | 19.079 | **<0.001** | 238.107 | **<0.001** |

HER2=human epidermal growth factor receptor 2. HR=hormone receptor. Luminal A=HER2 negative HR positive, and PR≥20% and Ki-67 <15%. Luminal B (HER2 negative) =HER2 negative HR positive, and PR < 20% or Ki-67≥15%. TNBC=triple-negative breast cancer (HR and HER2 negative-tumors). PR=progesterone receptor. Ki-67=proliferating cell nuclear antigen-67. BCS=[breast conserving surgery](https://pubmed.ncbi.nlm.nih.gov/31176055/). SM=simple mastectomy. SLNB=sentinel lymph node biopsy. ALND=axillary lymph node dissection.

**Table S7. Consensus Rate**

|  | **Consensus cases, n (%)** | **Non-consensus cases, n (%)** | **Chi-square test** | | **multivariate logistic regression** | |
| --- | --- | --- | --- | --- | --- | --- |
|  |  |  | ***χ*^2^** | ***P*** | **OR（95% Cl）** | ***P*** |
| **Overall** (N=2124) | 1363 (64.2) | 761 (35.8) |  |  |  |  |
| **Different Seniority of Physicians** |  |  | 36.974 | **<0.001** |  | **<0.001** |
| G1 (N=236) | 137 (58.1) | 99 (41.9) |  |  | 0.299 (0.193-0.462) | **<0.001** |
| G2 (N=236) | 153 (64.8) | 83 (35.2) |  |  | 0.394 (0.254-0.613) | **<0.001** |
| G3 (N=236) | 147 (62.3) | 89 (37.7) |  |  | 0.369 (0.239-0.572) | **<0.001** |
| G4 (N=236) | 138 (58.5) | 98 (41.5) |  |  | 0.290 (0.187-0.448) | **<0.001** |
| G5 (N=236) | 137 (58.1) | 99 (41.9) |  |  | 0.301 (0.195-0.466) | **<0.001** |
| G6 (N=236) | 154 (65.3) | 82 (34.7) |  |  | 0.400 (0.256-0.624) | **<0.001** |
| G7 (N=236) | 151 (64.0) | 85 (36.0) |  |  | 0.409 (0.263-0.637) | **<0.001** |
| G8 (N=236) | 158 (66.9) | 78 (33.1) |  |  | 0.477 (0.306-0.743) | **0.001** |
| G9 (N=236) | 188 (79.7) | 48 (20.3) |  |  | 1.000 (1.000-1.000) | Reference |
| **Age** |  |  | 5.620 | 0.060 |  | 0.393 |
| ≤44 (N=572) | 357 (62.4) | 215 (37.6) |  |  | 1.000 (1.000-1.000) | Reference |
| 45–54 (N=1020) | 642 (62.9) | 378 (37.1) |  |  | 0.949 (0.736-1.223) | 0.684 |
| ≥55 (N=532) | 364 (68.4) | 168 (31.6) |  |  | 1.157 (0.785-1.707) | 0.461 |
| **Menstrual status** |  |  | 2.139 | 0.144 |  | 0.518 |
| Premenopausal (N=1276) | 803 (62.9) | 473 (37.1) |  |  | 1.000 (1.000-1.000) | Reference |
| Postmenopausal (N=848) | 560 (66.0) | 288 (34.0) |  |  | 1.098 (0.827-1.457) | 0.518 |
| **Breast Surgery Modality** |  |  | 22.587 | **<0.001** |  | **<0.001** |
| BCS (N=384) | 206 (53.6) | 178 (46.4) |  |  | 1.000 (1.000-1.000) | Reference |
| SM (N=1740) | 1157 (66.5) | 583 (33.5) |  |  | 1.960 (1.490-2.578) | **<0.001** |
| **Axillary surgery modality** |  |  | 2.618 | 0.106 |  | 0.611 |
| ALND (N=1516) | 989 (65.2) | 527 (34.8) |  |  | 1.000 (1.000-1.000) | Reference |
| SLNB (N=608) | 374 (61.5) | 234 (38.5) |  |  | 1.067 (0.830-1.373) | 0.611 |
| **TNM Stage** |  |  | 1.307 | 0.520 |  | 0.570 |
| I (N=764) | 497 (65.1) | 267 (34.9) |  |  | 1.067 (0.768-1.482) | 0.700 |
| II (N=1060) | 668 (63.0) | 392 (37.0) |  |  | 0.947 (0.704-1.275) | 0.720 |
| **III (N=300)** | 198 (66.0) | 102 (34.0) |  |  | 1.000 (1.000-1.000) | Reference |
| **Molecular subtype** |  |  | 45.157 | **<0.001** |  | **<0.001** |
| HER2 positive HR negative (N=192) | 123 (64.1) | 69 (35.9) |  |  | 0.416 (0.267-0.647) | **<0.001** |
| HER2 positive HR positive (N=444) | 240 (54.1) | 204 (45.9) |  |  | 0.264 (0.183-0.381) | **<0.001** |
| Luminal A (N=324) | 214 (66.0) | 110 (34.0) |  |  | 0.436 (0.293-0.648) | **<0.001** |
| Luminal B (HER2 negative) (N=892) | 572 (64.1) | 320 (35.9) |  |  | 0.460 (0.328-0.646) | **<0.001** |
| TNBC (N=272) | 214 (78.7) | 58 (21.3) |  |  | 1.000 (1.000-1.000) | Reference |
| **Treatment Stage** |  |  | 141.955 | **<0.001** |  | **<0.001** |
| Adjuvant targeted therapy (N=531) | 442 (83.2) | 89 (16.8) |  |  | 1.000 (1.000-1.000) | Reference |
| Adjuvant chemotherapy (N=531) | 258 (48.6) | 273 (51.4) |  |  | 0.172 (0.128-0.231) | **<0.001** |
| Adjuvant radiotherapy (N=531) | 326 (61.4) | 205 (38.6) |  |  | 0.300 (0.224-0.404) | **<0.001** |
| Adjuvant endocrine therapy (N=531) | 337 (63.5) | 194 (36.5) |  |  | 0.330 (0.245-0.444) | **<0.001** |
| **Constant** |  |  |  |  | 16.494 | **<0.001** |

**Table S8. Decision Stability**

|  | **Decision stability case, n (%)** | **Decision instability case, n (%)** | **Chi-square test** | | **logistic regression-model 1** | | **logistic regression-model 2** | |
| --- | --- | --- | --- | --- | --- | --- | --- | --- |
|  |  |  | ***χ*^2^** | ***P*** | **OR（95% Cl）** | ***P*** | **OR（95% Cl）** | ***P*** |
| **Overall** (N=495) | 201 (40.6) | 294 (59.4) |  |  |  |  |  |  |
| **Different Seniority Physicians** |  |  | 2.613 | 0.271 |  | 0.117 |  | NA |
| **Senior Physician (N=165)** | 69 (41.8) | 96 (58.2) |  |  | 1.000 (1.000-1.000) | Ref | NA | NA |
| Middle Physician (N=165) | 73 (44.2) | 92 (55.8) |  |  | 1.004 (0.632-1.595) | 0.986 | NA | NA |
| Junior Physician (N=165) | 59 (35.8) | 106 (64.2) |  |  | 0.649 (0.404-1.043) | 0.074 | NA | NA |
| **Different Grade Hospitals** |  |  | 13.721 | **0.001** |  | **<0.001** |  | NA |
| Provincial Hospital (N=165) | 86 (52.1) | 79 (47.9) |  |  | 1.000 (1.000-1.000) | Ref | NA | NA |
| Municipal Hospital (N=165) | 56 (33.9) | 109 (66.1) |  |  | 0.391 (0.244-0.627) | **<0.001** | NA | NA |
| County Hospital (N=165) | 59 (35.8) | 106 (64.2) |  |  | 0.426 (0.267-0.681) | **<0.001** | NA | NA |
| **Different Seniority Physicians in Different Grades Hospitals** |  |  | 28.798 | **<0.001** |  | NA |  | **<0.001** |
| 301 Provincial Senior (N=55) | 29 (52.7) | 26 (47.3) |  |  | NA | NA | 0.974 (0.443-2.141) | 0.948 |
| 302 Provincial Middle (N=55) | 30 (54.5) | 25 (45.5) |  |  | NA | NA | 1.000 (1.000-1.000) | Ref |
| 303 Provincial Junior (N=55) | 27 (49.1) | 28 (50.9) |  |  | NA | NA | 0.656 (0.298-1.449) | 0.297 |
| 201 Municipal Senior (N=55) | 21 (38.2) | 34 (61.8) |  |  | NA | NA | 0.476 (0.214-1.059) | 0.069 |
| 202 Municipal Middle (N=55) | 14 (25.5) | 41 (74.5) |  |  | NA | NA | 0.207 (0.088-0.488) | **<0.001** |
| 203 Municipal Junior (N=55) | 21 (38.2) | 34 (61.8) |  |  | NA | NA | 0.372 (0.166-0.830) | **0.016** |
| 101 County Senior (N=55) | 19 (34.5) | 36 (65.5) |  |  | NA | NA | 0.353 (0.158-0.788) | **0.011** |
| 102 County Middle (N=55) | 29 (52.7) | 26 (47.3) |  |  | NA | NA | 0.751 (0.340-1.660) | 0.480 |
| 103 County Junior (N=55) | 11 (20.0) | 44 (80.0) |  |  | NA | NA | 0.167 (0.069-0.404) | **<0.001** |
| **Age** |  |  | 4.522 | 0.104 |  | 0.304 |  | 0.318 |
| ≤44 (N=141) | 55 (39.0) | 86 (61.0) |  |  | 1.000 (1.000-1.000) | Ref | 1.000 (1.000-1.000) | Ref |
| 45–54 (N=266) | 118 (44.4) | 148 (55.6) |  |  | 1.472 (0.898-2.411) | 0.125 | 1.472 (0.891-2.430) | 0.131 |
| ≥55 (N=88) | 28 (31.8) | 60 (68.2) |  |  | 1.387 (0.611-3.151) | 0.434 | 1.519 (0.662-3.486) | 0.324 |
| **Menstrual status** |  |  | 8.491 | **0.004** |  | **0.021** |  | **0.017** |
| Premenopausal (N=317) | 144 (45.4) | 173 (54.6) |  |  | 1.000 (1.000-1.000) | Ref | 1.000 (1.000-1.000) | Ref |
| Postmenopausal (N=178) | 57 (32.0) | 121 (68.0) |  |  | 0.502 (0.280-0.899) | **0.021** | 0.487 (0.270-0.877) | **0.017** |
| **Breast Surgery Modality** |  |  | 0.136 | 0.712 |  | 0.148 |  | 0.161 |
| BCS (N=120) | 47 (39.2) | 73 (60.8) |  |  | 1.000 (1.000-1.000) | Ref | 1.000 (1.000-1.000) | Ref |
| SM (N=375) | 154 (41.1) | 221 (58.9) |  |  | 1.507 (0.865-2.626) | 0.148 | 1.498 (0.852-2.635) | 0.161 |
| **Axillary surgery modality** |  |  | 0.023 | 0.879 |  | 0.391 |  | 0.372 |
| ALND (N=333) | 136 (40.8) | 197 (59.2) |  |  | 1.000 (1.000-1.000) | Ref | 1.000 (1.000-1.000) | Ref |
| SLNB (N=162) | 65 (40.1) | 97 (59.9) |  |  | 1.250 (0.751-2.081) | 0.391 | 1.267 (0.754-2.128) | 0.372 |
| **TNM Stage** |  |  | 3.553 | 0.169 |  | 0.249 |  | 0.253 |
| I (N=193) | 83 (43.0) | 110 (57.0) |  |  | 0.896 (0.423-1.901) | 0.775 | 0.815 (0.380-1.746) | 0.598 |
| II (N=247) | 91 (36.8) | 156 (63.2) |  |  | 0.660 (0.337-1.291) | 0.225 | 0.621 (0.314-1.231) | 0.172 |
| III (N=55) | 27 (49.1) | 28 (50.9) |  |  | 1.000 (1.000-1.000) | Ref | 1.000 (1.000-1.000) | Ref |
| **Molecular subtype** |  |  | 9.209 | 0.056 |  | 0.284 |  | 0.222 |
| HER2 positive HR negative (N=46) | 17 (37.0) | 29 (63.0) |  |  | 1.179 (0.433-3.208) | 0.747 | 1.157 (0.420-3.190) | 0.777 |
| HER2 positive HR positive (N=126) | 38 (30.2) | 88 (69.8) |  |  | 0.848 (0.359-2.001) | 0.706 | 0.778 (0.325-1.861) | 0.572 |
| Luminal A (N=66) | 31 (47.0) | 35 (53.0) |  |  | 1.634 (0.675-3.959) | 0.277 | 1.608 (0.653-3.957) | 0.301 |
| Luminal B (HER2 negative) (N=220) | 100 (45.5) | 120 (54.5) |  |  | 1.458 (0.676-3.145) | 0.336 | 1.416 (0.649-3.089) | 0.382 |
| **TNBC (N=37)** | 15 (40.5) | 22 (59.5) |  |  | 1.000 (1.000-1.000) | Ref | 1.000 (1.000-1.000) | Ref |
| **Treatment Stage** |  |  | 10.182 | **0.017** |  | 0.107 |  | 0.192 |
| Adjuvant targeted therapy (N=64) | 17 (26.6) | 47 (73.4) |  |  | 1.000 (1.000-1.000) | Ref | 1.000 (1.000-1.000) | Ref |
| Adjuvant chemotherapy (N=214) | 81 (37.9) | 133 (62.1) |  |  | 1.218 (0.582-2.547) | 0.601 | 1.265 (0.600-2.669) | 0.537 |
| Adjuvant radiotherapy (N=109) | 51 (46.8) | 58 (53.2) |  |  | 2.322 (1.012-5.325) | **0.047** | 2.197 (0.944-5.110) | 0.068 |
| Adjuvant endocrine therapy (N=108) | 52 (48.1) | 56 (51.9) |  |  | 1.614 (0.711-3.665) | 0.252 | 1.666 (0.727-3.817) | 0.228 |
| **常数项（Constant）** |  |  |  |  | 0.660 | 0.553 | 0.725 | 0.666 |

Ref=Reference. NA=not applicable. HER2=human epidermal growth factor receptor 2. HR=hormone receptor. Luminal A=HER2 negative HR positive, and PR≥20% and Ki-67 <15%. Luminal B (HER2 negative) =HER2 negative HR positive, and PR < 20% or Ki-67≥15%. TNBC=triple-negative breast cancer (HR and HER2 negative-tumors). PR=progesterone receptor. Ki-67=proliferating cell nuclear antigen-67. BCS=[breast conserving surgery](https://pubmed.ncbi.nlm.nih.gov/31176055/). SM=simple mastectomy. SLNB=sentinel lymph node biopsy. ALND=axillary lymph node dissection.

**Table S9. Guideline Conformity**

|  | **Overall physician-guideline conformity (before calibration)** | | | **Overall physicians-guideline conformity (after calibration)** | | | **CSCO AI-guideline conformity** | | | **Overall physicians *vs.* CSCO AI** |
| --- | --- | --- | --- | --- | --- | --- | --- | --- | --- | --- |
|  | **Conformity cases, n (%)** | **Non-conformity cases, n (%)** | ***P*** | **Conformity cases, n (%)** | **Non-conformity cases, n (%)** | ***P*** | **Conformity cases, n (%)** | **Non-conformity cases, n (%)** | ***P*** | ***P*** |
| **Overall** (N=6372) | 5100 (80.0) | 1272 (20.0) |  | 5100 (80.0) | 1272 (20.0) |  | 6213 (97.5) | 159 (2.5) |  | **<0.001** |
| **Different Seniority Physicians** |  |  | 0.648 |  |  | **<0.001** |  |  | 1.000 |  |
| Senior Physician (N=2124) | 1713 (80.6) | 411 (19.4) |  | 1713 (80.6) | 411 (19.4) |  | 2071 (97.5) | 53 (2.5) |  | **<0.001** |
| Middle Physician (N=2124) | 1689 (79.5) | 435 (20.5) |  | 1786 (84.1) | 338 (15.9) |  | 2071 (97.5) | 53 (2.5) |  | **<0.001** |
| Junior Physician (N=2124) | 1698 (79.9) | 426 (20.1) |  | 1454 (68.5) | 670 (31.5) |  | 2071 (97.5) | 53 (2.5) |  | **<0.001** |
| **Different Grade Hospitals** |  |  | **0.009** |  |  | **<0.001** |  |  | 1.000 |  |
| Provincial Hospital (N=2124) | 1662 (78.2) | 462 (21.8) |  | 1662 (78.2) | 462 (21.8) |  | 2071 (97.5) | 53 (2.5) |  | **<0.001** |
| Municipal Hospital (N=2124) | 1696 (79.8) | 428 (20.2) |  | 1104 (52.0) | 1020 (48.0) |  | 2071 (97.5) | 53 (2.5) |  | **<0.001** |
| County Hospital (N=2124) | 1742 (82.0) | 382 (18.0) |  | 1197 (56.4) | 927 (43.6) |  | 2071 (97.5) | 53 (2.5) |  | **<0.001** |
| **Different Seniority Physicians in Different Grades Hospitals** |  |  | **<0.001** |  |  | **<0.001** |  |  | 0.357 |  |
| 301 Provincial Senior (N=708) | 592 (83.6) | 116 (16.4) |  | 572 (80.9) | 136 (19.1) |  | 691 (97.6) | 17 (2.4) |  | **<0.001** |
| 302 Provincial Middle (N=708) | 544 (76.8) | 164 (23.2) |  | 544 (76.8) | 164 (23.2) |  | 685 (96.8) | 23 (3.2) |  | **<0.001** |
| 303 Provincial Junior (N=708) | 526 (74.3) | 182 (25.7) |  | 474 (66.9) | 234 (33.1) |  | 695 (98.2) | 13 (1.8) |  | **<0.001** |
| 201 Municipal Senior (N=708) | 556 (78.5) | 152 (21.5) |  | 390 (55.0) | 318 (45.0) |  | 695 (98.2) | 13 (1.8) |  | **<0.001** |
| 202 Municipal Middle (N=708) | 574 (81.1) | 134 (18.9) |  | 269 (37.9) | 439 (62.1) |  | 691 (97.6) | 17 (2.4) |  | **<0.001** |
| 203 Municipal Junior (N=708) | 566 (79.9) | 142 (20.1) |  | 397 (56.0) | 311 (44.0) |  | 685 (96.8) | 23 (3.2) |  | **<0.001** |
| 101 County Senior (N=708) | 565 (79.8) | 143 (20.2) |  | 358 (50.5) | 350 (49.5) |  | 685 (96.8) | 23 (3.2) |  | **<0.001** |
| 102 County Middle (N=708) | 571 (80.6) | 137 (19.4) |  | 552 (78.0) | 156 (22.0) |  | 695 (98.2) | 13 (1.8) |  | **<0.001** |
| 103 County Junior (N=708) | 606 (85.6) | 102 (14.4) |  | 222 (31.4) | 486 (68.6) |  | 691 (97.6) | 17 (2.4) |  | **<0.001** |
| **Physicians** |  |  | **<0.001** |  |  | NA |  |  | **0.023** |  |
| 3011 Provincial Senior1 (N=236) | 180 (76.3) | 56 (23.7) |  | NA | NA |  | 229 (97.0) | 7 (3.0) |  | **<0.001** |
| 3012 Provincial Senior2 (N=236) | 217 (91.9) | 19 (8.1) |  | NA | NA |  | 235 (99.6) | 1 (0.4) |  | **<0.001** |
| 3013 Provincial Senior3 (N=236) | 195 (82.6) | 41 (17.4) |  | NA | NA |  | 227 (96.2) | 9 (3.8) |  | **<0.001** |
| 3021 Provincial Middle1 (N=236) | 203 (86.0) | 33 (14.0) |  | NA | NA |  | 225 (95.3) | 11 (4.7) |  | **0.001** |
| 3022 Provincial Middle2 (N=236) | 161 (68.2) | 75 (31.8) |  | NA | NA |  | 232 (98.3) | 4 (1.7) |  | **<0.001** |
| 3023 Provincial Middle3 (N=236) | 180 (76.3) | 56 (23.7) |  | NA | NA |  | 228 (96.6) | 8 (3.4) |  | **<0.001** |
| 3031 Provincial Junior1 (N=236) | 189 (80.1) | 47 (19.9) |  | NA | NA |  | 230 (97.5) | 6 (2.5) |  | **<0.001** |
| 3032 Provincial Junior2 (N=236) | 177 (75.0) | 59 (25.0) |  | NA | NA |  | 232 (98.3) | 4 (1.7) |  | **<0.001** |
| 3034 Provincial Junior4 (N=236) | 160 (67.8) | 76 (32.2) |  | NA | NA |  | 233 (98.7) | 3 (1.3) |  | **<0.001** |
| 2011 Municipal Senior1 (N=236) | 200 (84.7) | 36(15.3) |  | NA | NA |  | 232 (98.3) | 4 (1.7) |  | **<0.001** |
| 2012 Municipal Senior2 (N=236) | 195 (82.6) | 41 (17.4) |  | NA | NA |  | 230 (97.5) | 6 (2.5) |  | **<0.001** |
| 2013 Municipal Senior3 (N=236) | 161 (68.2) | 75 (31.8) |  | NA | NA |  | 233 (98.7) | 3 (1.3) |  | **<0.001** |
| 2021 Municipal Middle1 (N=236) | 193 (81.8) | 43 (18.2) |  | NA | NA |  | 227 (96.2) | 9 (3.8) |  | **<0.001** |
| 2022 Municipal Middle2 (N=236) | 177 (75.0) | 59 (25.0) |  | NA | NA |  | 229 (97.0) | 7 (3.0) |  | **<0.001** |
| 2023 Municipal Middle3 (N=236) | 204 (86.4) | 32 (13.6) |  | NA | NA |  | 235 (99.6) | 1 (0.4) |  | **<0.001** |
| 2031 Municipal Junior1 (N=236) | 210 (89.0) | 26 (11.0) |  | NA | NA |  | 228 (96.6) | 8 (3.4) |  | **<0.001** |
| 2032 Municipal Junior2 (N=236) | 147 (62.3) | 89 (37.7) |  | NA | NA |  | 225 (95.3) | 11 (4.7) |  | **<0.001** |
| 2033 Municipal Junior3 (N=236) | 209 (88.6) | 27 (11.4) |  | NA | NA |  | 232 (98.3) | 4 (1.7) |  | **<0.001** |
| 1011 County Senior1 (N=236) | 172 (72.9) | 64 (27.1) |  | NA | NA |  | 228 (96.6) | 8 (3.4) |  | **<0.001** |
| 1012 County Senior2 (N=236) | 183 (77.5) | 53 (22.5) |  | NA | NA |  | 232 (98.3) | 4 (1.7) |  | **<0.001** |
| 1013 County Senior3 (N=236) | 210 (89.0) | 26 (11.0) |  | NA | NA |  | 225 (95.3) | 11 (4.7) |  | **0.006** |
| 1021 County Middle1 (N=236) | 212 (89.8) | 24 (10.2) |  | NA | NA |  | 230 (97.5) | 6 (2.5) |  | **<0.001** |
| 1022 County Middle2 (N=236) | 175 (74.2) | 61 (25.8) |  | NA | NA |  | 232 (98.3) | 4 (1.7) |  | **<0.001** |
| 1023 County Middle3 (N=236) | 184 (78.0) | 52 (22.0) |  | NA | NA |  | 233 (98.7) | 3 (1.3) |  | **<0.001** |
| 1031 County Junior1 (N=236) | 192 (81.4) | 44 (18.6) |  | NA | NA |  | 227 (96.2) | 9 (3.8) |  | **<0.001** |
| 1032 County Junior2 (N=236) | 219 (92.8) | 17 (7.2) |  | NA | NA |  | 235 (99.6) | 1 (0.4) |  | **<0.001** |
| 1033 County Junior3 (N=236) | 195 (82.6) | 41 (17.4) |  | NA | NA |  | 229 (97.0) | 7 (3.0) |  | **<0.001** |
| **Age** |  |  | **0.039** |  |  | **<0.001** |  |  | **0.016** |  |
| ≤44 (N=1716) | 1377 (80.2) | 339 (19.8) |  | 1210 (70.5) | 506 (29.5) |  | 1677 (97.7) | 39 (2.3) |  | **<0.001** |
| 45–54 (N=3060) | 2414 (78.9) | 646 (21.1) |  | 2414 (78.9) | 646 (21.1) |  | 2967 (97.0) | 93 (3.0) |  | **<0.001** |
| ≥55 (N=1596) | 1309 (82.0) | 287 (18.0) |  | 938 (58.7) | 658 (41.3) |  | 1569 (98.3) | 27 (1.7) |  | **<0.001** |
| **Menstrual status** |  |  | 0.127 |  |  | **<0.001** |  |  | 0.937 |  |
| Premenopausal (N=3828) | 3040 (79.4) | 788 (20.6) |  | 3040 (79.4) | 788 (20.6) |  | 3732 (97.5) | 96 (2.5) |  | **<0.001** |
| Postmenopausal (N=2544) | 2060 (81.0) | 484 (19.0) |  | 1452 (57.1) | 1092 (42.9) |  | 2481 (97.5) | 63 (2.5) |  | **<0.001** |
| **Breast Surgery Modality** |  |  | **<0.001** |  |  | **<0.001** |  |  | 0.130 |  |
| BCS (N=1152) | 843 (73.2) | 309 (26.8) |  | 804 (69.8) | 348 (30.2) |  | 1116 (96.9) | 36 (3.1) |  | **<0.001** |
| SM (N=5220) | 4257 (81.6) | 963 (18.4) |  | 4257 (81.6) | 963 (18.4) |  | 5097 (97.6) | 123 (2.4) |  | **<0.001** |
| **Axillary surgery modality** |  |  | **<0.001** |  |  | **<0.001** |  |  | 0.659 |  |
| ALND (N=4548) | 3694 (81.2) | 854 (18.8) |  | 3694 (81.2) | 854 (18.8) |  | 4437 (97.6) | 111 (2.4) |  | **<0.001** |
| SLNB (N=1824) | 1406 (77.1) | 418 (22.9) |  | 1382 (75.8) | 442 (24.2) |  | 1776 (97.4) | 48 (2.6) |  | **<0.001** |
| **TNM Stage** |  |  | **0.004** |  |  | **<0.001** |  |  | **<0.001** |  |
| I (N=2292) | 1794 (78.3) | 498 (21.7) |  | 1571 (68.5) | 721 (31.5) |  | 2220 (96.9) | 72 (3.1) |  | **<0.001** |
| II (N=3180) | 2555 (80.3) | 625 (19.7) |  | 1915 (60.2) | 1265 (39.8) |  | 3096 (97.4) | 84 (2.6) |  | **<0.001** |
| III (N=900) | 751 (83.4) | 149 (16.6) |  | 751 (83.4) | 149 (16.6) |  | 897 (99.7) | 3 (0.3) |  | **<0.001** |
| **Molecular subtype** |  |  | **<0.001** |  |  | **<0.001** |  |  | **<0.001** |  |
| HER2 positive HR negative (N=576) | 455 (79.0) | 121 (21.0) |  | 370 (64.2) | 206 (35.8) |  | 546 (94.8) | 30 (5.2) |  | **<0.001** |
| HER2 positive HR positive (N=1332) | 987 (74.1) | 345 (25.9) |  | 655 (49.2) | 677 (50.8) |  | 1287 (96.6) | 45 (3.4) |  | **<0.001** |
| Luminal A (N=972) | 808 (83.1) | 164 (16.9) |  | 835 (85.9) | 137 (14.1) |  | 936 (96.3) | 36 (3.7) |  | **<0.001** |
| Luminal B (HER2 negative) (N=2676) | 2134 (79.7) | 542 (20.3) |  | 2134 (79.7) | 542 (20.3) |  | 2634 (98.4) | 42 (1.6) |  | **<0.001** |
| TNBC (N=816) | 716 (87.7) | 100 (12.3) |  | 637 (78.1) | 179 (21.9) |  | 810 (99.3) | 6 (0.7) |  | **<0.001** |
| **Treatment Stage** |  |  | **<0.001** |  |  | **<0.001** |  |  | **<0.001** |  |
| Adjuvant targeted therapy (N=1593) | 1431 (89.8) | 162 (10.2) |  | 791 (49.7) | 802 (50.3) |  | 1563 (98.1) | 30 (1.9) |  | **<0.001** |
| Adjuvant chemotherapy (N=1593) | 1075 (67.5) | 518 (32.5) |  | 847 (53.2) | 746 (46.8) |  | 1512 (94.9) | 81 (5.1) |  | **<0.001** |
| Adjuvant radiotherapy (N=1593) | 1279 (80.3) | 314 (19.7) |  | 1244 (78.1) | 349 (21.9) |  | 1590 (99.8) | 3 (0.2) |  | **<0.001** |
| Adjuvant endocrine therapy (N=1593) | 1315 (82.5) | 278 (17.5) |  | 1315 (82.5) | 278 (17.5) |  | 1548 (97.2) | 45 (2.8) |  | **<0.001** |

**NA=not applicable. HER2=human epidermal growth factor receptor 2. HR=hormone receptor. Luminal A=HER2 negative HR positive, and PR≥20% and Ki-67 <15%. Luminal B (HER2 negative) =HER2 negative HR positive, and PR < 20% or Ki-67≥15%. TNBC=triple-negative breast cancer (HR and HER2 negative-tumors). PR=progesterone receptor. Ki-67=proliferating cell nuclear antigen-67. BCS=**[**breast conserving surgery**](https://pubmed.ncbi.nlm.nih.gov/31176055/)**. SM=simple mastectomy. SLNB=sentinel lymph node biopsy. ALND=axillary lymph node dissection. Table S10.The Logistic Regression Analysis of Guideline Conformity**

|  | **Overall physicians-guideline conformity-model 1** | | **Overall physicians-guideline conformity-model 2** | | **CSCO AI-guideline conformity-model 1** | | **CSCO AI-guideline conformity-model 2** | |
| --- | --- | --- | --- | --- | --- | --- | --- | --- |
|  | **OR（95% Cl）** | ***P*** | **OR（95% Cl）** | ***P*** | **OR（95% Cl）** | ***P*** | **OR（95% Cl）** | ***P*** |
| **Overall** (N=6372) |  |  |  |  |  |  |  |  |
| **Different Seniority Physicians** |  | 0.621 |  | NA |  | 1.000 |  | NA |
| Senior Physician (N=2124) | 1.000 (1.000-1.000) | Reference | NA | NA | 1.000 (1.000-1.000) | Reference | NA | NA |
| Middle Physician (N=2124) | 0.926 (0.793-1.082) | 0.334 | NA | NA | 1.000 (0.675-1.481) | 1.000 | NA | NA |
| Junior Physician (N=2124) | 0.953 (0.815-1.114) | 0.543 | NA | NA | 1.000 (0.675-1.481) | 1.000 | NA | NA |
| **Different Grade Hospitals** |  | **0.006** |  | NA |  | 1.000 |  | NA |
| Provincial Hospital (N=2124) | 1.000 (1.000-1.000) | Reference | NA | NA | 1.000 (1.000-1.000) | Reference | NA | NA |
| Municipal Hospital (N=2124) | 1.109 (0.952-1.292) | 0.184 | NA | NA | 1.000 (0.675-1.481) | 1.000 | NA | NA |
| County Hospital (N=2124) | 1.288 (1.102-1.506) | **0.001** | NA | NA | 1.000 (0.675-1.481) | 1.000 | NA | NA |
| **Different Seniority Physicians in Different Grades Hospitals** |  | NA |  | **<0.001** |  | NA |  | 0.305 |
| 301 Provincial Senior (N=708) | NA | NA | 1.000 (1.000-1.000) | Reference | NA | NA | 1.000 (1.000-1.000) | Reference |
| 302 Provincial Middle (N=708) | NA | NA | 0.609 (0.463-0.801) | **<0.001** | NA | NA | 0.658 (0.343-1.263) | 0.208 |
| 303 Provincial Junior (N=708) | NA | NA | 0.539 (0.411-0.707) | **<0.001** | NA | NA | 1.193 (0.566-2.514) | 0.643 |
| 201 Municipal Senior (N=708) | NA | NA | 0.695 (0.527-0.918) | **0.010** | NA | NA | 1.193 (0.566-2.514) | 0.643 |
| 202 Municipal Middle (N=708) | NA | NA | 0.830 (0.626-1.101) | 0.196 | NA | NA | 1.000 (0.500-1.999) | 1.000 |
| 203 Municipal Junior (N=708) | NA | NA | 0.741 (0.560-0.981) | **0.036** | NA | NA | 0.658 (0.343-1.263) | 0.208 |
| 101 County Senior (N=708) | NA | NA | 0.734 (0.555-0.971) | **0.030** | NA | NA | 0.658 (0.343-1.263) | 0.208 |
| 102 County Middle (N=708) | NA | NA | 0.800 (0.603-1.061) | 0.121 | NA | NA | 1.193 (0.566-2.514) | 0.643 |
| 103 County Junior (N=708) | NA | NA | 1.174 (0.872-1.580) | 0.290 | NA | NA | 1.000 (0.500-1.999) | 1.000 |
| **Age** |  | 0.080 |  | 0.113 |  | **0.008** |  | **0.005** |
| ≤44 (N=1716) | 1.000 (1.000-1.000) | Reference | 1.000 (1.000-1.000) | Reference | 1.000 (1.000-1.000) | Reference | 1.000 (1.000-1.000) | Reference |
| 45–54 (N=3060) | 0.844 (0.713-0.999) | **0.049** | 0.867 (0.732-1.028) | 0.100 | 0.866 (0.561-1.339) | 0.518 | 0.872 (0.564-1.348) | 0.537 |
| ≥55 (N=1596) | 0.945 (0.729-1.224) | 0.666 | 0.990 (0.763-1.285) | 0.941 | 1.897 (0.976-3.687) | 0.059 | 1.985 (1.021-3.863) | **0.043** |
| **Menstrual status** |  | 0.270 |  | 0.381 |  | 0.251 |  | 0.218 |
| Premenopausal (N=3828) | 1.000 (1.000-1.000) | Reference | 1.000 (1.000-1.000) | Reference | 1.000 (1.000-1.000) | Reference | 1.000 (1.000-1.000) | Reference |
| Postmenopausal (N=2544) | 1.111 (0.922-1.338) | 0.270 | 1.087 (0.902-1.311) | 0.381 | 0.778 (0.506-1.195) | 0.251 | 0.763 (0.497-1.173) | 0.218 |
| **Breast Surgery Modality** |  | **<0.001** |  | **<0.001** |  | **0.006** |  | **0.004** |
| BCS (N=1152) | 1.000 (1.000-1.000) | Reference | 1.000 (1.000-1.000) | Reference | 1.000 (1.000-1.000) | Reference | 1.000 (1.000-1.000) | Reference |
| SM (N=5220) | 1.680 (1.411-2.000) | **<0.001** | 1.750 (1.468-2.088) | **<0.001** | 1.883 (1.195-2.968) | **0.006** | 1.979 (1.249-3.136) | **0.004** |
| **Axillary surgery modality** |  | 0.940 |  | 0.609 |  | **0.018** |  | **0.019** |
| ALND (N=4548) | 1.000 (1.000-1.000) | Reference | 1.000 (1.000-1.000) | Reference | 1.000 (1.000-1.000) | Reference | 1.000 (1.000-1.000) | Reference |
| SLNB (N=1824) | 1.006 (0.854-1.186) | 0.940 | 1.044 (0.885-1.232) | 0.609 | 1.661 (1.091-2.530) | **0.018** | 1.665 (1.089-2.546) | **0.019** |
| **TNM Stage** |  | **0.007** |  | **0.011** |  | **<0.001** |  | **<0.001** |
| I (N=2292) | 0.706 (0.565-0.882) | **0.002** | 0.712 (0.569-0.890) | **0.003** | 0.081 (0.025-0.262) | **<0.001** | 0.080 (0.025-0.261) | **<0.001** |
| II (N=3180) | 0.818 (0.666-1.004) | 0.055 | 0.811 (0.659-0.997) | **0.047** | 0.108 (0.034-0.344) | **<0.001** | 0.110 (0.034-0.354) | **<0.001** |
| III (N=900) | 1.000 (1.000-1.000) | Reference | 1.000 (1.000-1.000) | Reference | 1.000 (1.000-1.000) | Reference | 1.000 (1.000-1.000) | Reference |
| **Molecular subtype** |  | **<0.001** |  | **<0.001** |  | **<0.001** |  | **<0.001** |
| HER2 positive HR negative (N=576) | 0.487 (0.361-0.657) | **<0.001** | 0.466 (0.345-0.630) | **<0.001** | 0.128 (0.052-0.314) | **<0.001** | 0.128 (0.052-0.314) | **<0.001** |
| HER2 positive HR positive (N=1332) | 0.367 (0.286-0.471) | **<0.001** | 0.359 (0.280-0.462) | **<0.001** | 0.201 (0.085-0.477) | **<0.001** | 0.200 (0.084-0.474) | **<0.001** |
| Luminal A (N=972) | 0.703 (0.533-0.928) | **0.013** | 0.675 (0.511-0.891) | **0.006** | 0.197 (0.082-0.476) | **<0.001** | 0.200 (0.083-0.483) | **<0.001** |
| Luminal B (HER2 negative) (N=2676) | 0.538 (0.425-0.680) | **<0.001** | 0.528 (0.417-0.668) | **<0.001** | 0.482 (0.203-1.146) | 0.099 | 0.488 (0.205-1.160) | 0.104 |
| TNBC (N=816) | 1.000 (1.000-1.000) | Reference | 1.000 (1.000-1.000) | Reference | 1.000 (1.000-1.000) | Reference | 1.000 (1.000-1.000) | Reference |
| **Treatment Stage** |  | **<0.001** |  | **<0.001** |  | **<0.001** |  | **<0.001** |
| Adjuvant targeted therapy (N=1593) | 1.000 (1.000-1.000) | Reference | 1.000 (1.000-1.000) | Reference | 1.000 (1.000-1.000) | Reference | 1.000 (1.000-1.000) | Reference |
| Adjuvant chemotherapy (N=1593) | 0.227 (0.187-0.276) | **<0.001** | 0.225 (0.185-0.274) | **<0.001** | 0.351 (0.229-0.539) | **<0.001** | 0.350 (0.228-0.537) | **<0.001** |
| Adjuvant radiotherapy (N=1593) | 0.454 (0.370-0.558) | **<0.001** | 0.453 (0.368-0.556) | **<0.001** | 10.286 (3.129-33.818) | **<0.001** | 10.302 (3.133-33.878) | **<0.001** |
| Adjuvant endocrine therapy (N=1593) | 0.529 (0.429-0.653) | **<0.001** | 0.528 (0.428-0.651) | **<0.001** | 0.656 (0.410-1.051) | 0.079 | 0.656 (0.409-1.050) | 0.079 |
| **Constant** | 13.527 | **<0.001** | 18.526 | **<0.001** | 898.809 | **<0.001** | 945.861 | **<0.001** |

NA=not applicable. HER2=human epidermal growth factor receptor 2. HR=hormone receptor. Luminal A=HER2 negative HR positive, and PR≥20% and Ki-67 <15%. Luminal B (HER2 negative) =HER2 negative HR positive, and PR < 20% or Ki-67≥15%. TNBC=triple-negative breast cancer (HR and HER2 negative-tumors). PR=progesterone receptor. Ki-67=proliferating cell nuclear antigen-67. BCS=[breast conserving surgery](https://pubmed.ncbi.nlm.nih.gov/31176055/). SM=simple mastectomy. SLNB=sentinel lymph node biopsy. ALND=axillary lymph node dissection.

**Table S11. The Specific Cases and Reasons for Nonconformity with the Guidelines in Different Treatment Stages.**

| **Treatment Stages** | **Non-conformity Situation** | **Specific Cases and Decisions** | **Reasons** |
| --- | --- | --- | --- |
| Adjuvant Targeted Therapy | HER-2+,T1N0M0, without other risk factors | **[Case One: 22†]** Patient, female, 65 years old, postmenopausal. BUS: left breast mass 1.0*1.1 cm. 2016-1-4 Left breast mass VAB showed: invasive carcinoma, non-specific type, WHO grade II, nerve invasion (+). ER (80%), PR (1%), HER-2 (2+/FISH+), KI-67(20%). 2016-01-08 " Left breast cancer breast-conserving surgery + ALND" showed: ALN (0/8).  **[Decision]** Guidelines TC+H *vs.* CDSS HP | Studies have shown that patients with small tumors of HER2+ , LN- still have a higher risk of recurrence compared to patients with small tumors of HER2-;^4^ for these patients, further chemotherapy can be added to trastuzumab; studies showed that early breast cancer patients with TC+H regimen had 2-year DFS and OS rates of 97.8% and 99.2%.^4^ Therefore, the TC+H regimen can be considered for low-risk patients with T1N0, HER-2-positive disease. This opinion was jointly endorsed by two experts in this trial guideline experts group. |
| Adjuvant Chemotherapy | Luminal A, 1-3 positive axillary lymph nodes and with other recurrence risks | **[Case Two: 485†]** Patient, female, 54 years old, postmenopausal. 2020-7-16 right breast cancer modified radical surgery showed: cancer size 2.5*2.0*1.0cm, invasive carcinoma, non-specific type, WHO grade II; ALN (1+/27). ER (2-3+, 95%), PR (1+, 5%), Her-2 (1+), Ki-67 (12%) . pT2N1M0 Stage IIB Luminal A  **[ Decision ]** Guidelines AC/TC *vs.* CDSS AC-T/ddAC-T | Most experts consider that Luminal A breast cancer responds poorly to chemotherapy; AC-T regimens are recommended in the first place only for high-risk patients with >4 positive lymph nodes; whereas for other patients with indicators that require chemotherapy (including LN1-3 positive, ≥T2, G3, age <35 years, and ALN1-3 positive with other recurrence risks, etc.) the AC or TC regimen was recommended as a first choice. This opinion was jointly endorsed by the three experts of this trial expert group. |
| Adjuvant Radiotherapy | LuminalB(HER-2-), T1bN0M0, G1, Ki-67≥15%, premenopausal | **[Case Three: 46†]** Patient, female, 52 years old, premenopausal. BUS: right breast mass 0.7*0.8*0.7 cm. 2014-11-12 right breast mass VAB showed: invasive carcinoma, non-specific type, WHO grade I, nerve invasion (+). ER (3+, >90%),PR (-), HER-2 (1+), ki-67 (20%). 2014-11-28 right breast cancer modified radical surgery showed: excavated area with no cancer residue seen; ALN (0/22). pT1bN0M0 Stage IA Luminal B (HER-2 negative) type  **[ Decision ]** Guidelines TAM *vs.* CDSS OFS+TAM | The patients included in the postoperative unadjuvant chemotherapy subgroup of the SOFT study^5^ were mostly LN-, G1, and T≤2 cm; subgroup analysis showed limited benefit from OFS combined with endocrine therapy in such patients. Therefore, the CSCO guidelines recommend a basic strategy of adjuvant postoperative endocrine therapy with TAM for 5 years for patients with low recurrence risk who fulfill the conditions of both lymph node negativity, G1, T≤2 cm and low Ki-67 expression (<15%). For patients with Ki-67≥15%, the guidelines are not made clear, and the TAM regimen is still recommended by the guideline expert group of this trial. This opinion was jointly endorsed by two experts in this trial guideline panel. The other expert believed that either TAM or OFS+TAM was acceptable. |
| Adjuvant Endocrine Therapy | Incorrect identification of case information | **[Case Four: 154†]** Patient, female, 28 years old, premenopausal. 2019-12-09 left breast cancer breast-conserving surgery + ALND showed: cancer size 2.5*1.5*1cm, invasive carcinoma, non-specific type, WHO grade III, lymphovascular tumor thrombus (+). er (80%), pr (70%), HER-2 (3+), KI-67 (+, 15%),. ALN (3+/15). pT2N1M0  Stage IIB Luminal B (HER-2-positive) type  **[Decision]** Guidelines Whole breast radiotherapy + tumor bed addition + regional lymph node radiotherapy  *vs.* CDSS Whole breast radiotherapy + tumor bed addition + regional lymph node radiotherapy including axilla | The true case was breast-conserving + ALND; CSCO AI incorrectly identified it as breast-conserving + SLNB. |

**†** The number is the relevant case' ID number. HER2=human epidermal growth factor receptor 2. HR=hormone receptor. Luminal A=HER2 negative HR positive, and PR≥20% and Ki-67 <15%. Luminal B (HER2 negative) =HER2 negative HR positive, and PR < 20% or Ki-67≥15%. TNBC=triple-negative breast cancer (HR and HER2 negative-tumors). PR=progesterone receptor. Ki-67=proliferating cell nuclear antigen-67. SLNB=sentinel lymph node biopsy. ALND=axillary lymph node dissection. CSCO AI= the Chinese Society of Clinical Oncology Artificial Intelligence System. AC: anthracycline combined with cyclophosphamide. TC=docetaxel combined with cyclophosphamide. AC-T=anthracycline combined with cyclophosphamide sequential paclitaxel. TAM=Tamoxifen. OFS=ovarian function suppression.

**Supplementary References**

1. An X, Lei X, Huang R, et al. Adjuvant chemotherapy for small, lymph node-negative, triple-negative breast cancer: A single-center study and a meta-analysis of the published literature. *Cancer* 2020; **126 Suppl 16**: 3837-46.

2. Ren YX, Hao S, Jin X, et al. Effects of adjuvant chemotherapy in T1N0M0 triple-negative breast cancer. *Breast (Edinburgh, Scotland)* 2019; **43**: 97-104.

3. Zhong W, Tan L, Jiang WG, et al. Effect of younger age on survival outcomes in T1N0M0 breast cancer: A propensity score matching analysis. *Journal of surgical oncology* 2019; **119**(8): 1039-46.

4. Jones SE, Collea R, Paul D, et al. Adjuvant docetaxel and cyclophosphamide plus trastuzumab in patients with HER2-amplified early stage breast cancer: a single-group, open-label, phase 2 study. *The Lancet Oncology* 2013; **14**(11): 1121-8.

5. Francis PA, Pagani O, Fleming GF, et al. Tailoring Adjuvant Endocrine Therapy for Premenopausal Breast Cancer. *The New England journal of medicine* 2018; **379**(2): 122-37.
